# RNAi-dependent heterochromatin assembly in fission yeast *Schizosaccharomyces pombe* requires heat-shock molecular chaperones Hsp90 and Mas5

**DOI:** 10.1186/s13072-018-0199-8

**Published:** 2018-06-04

**Authors:** Kosuke Okazaki, Hiroaki Kato, Tetsushi Iida, Kaori Shinmyozu, Jun-ichi Nakayama, Yota Murakami, Takeshi Urano

**Affiliations:** 1 Department of Biochemistry, Shimane University School of Medicine, 89-1 Enya-cho, Izumo, Shimane 693-8501 Japan; 2 Division of Cytogenetics, National Institute of Genetics, Mishima, 1111 Yata, Mishima, 411-8540 Japan; 3 Proteomics Support Unit, RIKEN Center for Developmental Biology, Kobe, Hyogo 650-0047 Japan; 4 Division of Chromatin Regulation, National Institute for Basic Biology, Okazaki, Aichi 444-8585 Japan; 5 Department of Chemistry, Faculty of Science, Hokkaido University, Sapporo, Hokkaido 060-0810 Japan; 6 Present Address: KNC Laboratories Co. Ltd., Kobe, Hyogo 651-2271 Japan; 7 Present Address: Laboratory for Genome Regeneration, Institute for Quantitative Biosciences, The University of Tokyo, Bunkyo-ku, Tokyo, 113-0032 Japan; 8 Present Address: National Cerebral and Cardiovascular Center, Suita, Osaka 565-8565 Japan

**Keywords:** RNAi, Heterochromatin, Fission yeast, *Schizosaccharomyces pombe*, Heat-shock molecular chaperons, Hsp90, Mas5

## Abstract

**Background:**

Heat-shock molecular chaperone proteins (Hsps) promote the loading of small interfering RNA (siRNA) onto RNA interference (RNAi) effector complexes. While the RNAi process is coupled with heterochromatin assembly in several model organisms, it remains unclear whether the Hsps contribute to epigenetic gene regulation. In this study, we used the fission yeast *Schizosaccharomyces pombe* as a model organism and investigated the roles of Hsp90 and Mas5 (a nucleocytoplasmic type-I Hsp40 protein) in RNAi-dependent heterochromatin assembly.

**Results:**

Using a genetic screen and biochemical analyses, we identified Hsp90 and Mas5 as novel silencing factors. Mutations in the genes encoding these factors caused derepression of silencing at the pericentromere, where heterochromatin is assembled in an RNAi-dependent manner, but not at the subtelomere, where RNAi is dispensable. The mutations also caused a substantial reduction in the level of dimethylation of histone H3 at Lys9 at the pericentromere, where association of the Argonaute protein Ago1 was also abrogated. Consistently, siRNA corresponding to the pericentromeric repeats was undetectable in these mutant cells. In addition, levels of Tas3, which is a protein in the RNA-induced transcriptional silencing complex along with Ago1, were reduced in the absence of Mas5.

**Conclusions:**

Our results suggest that the Hsps Hsp90 and Mas5 contribute to RNAi-dependent heterochromatin assembly. In particular, Mas5 appears to be required to stabilize Tas3 in vivo. We infer that impairment of Hsp90 and Hsp40 also may affect the integrity of the epigenome in other organisms.

**Electronic supplementary material:**

The online version of this article (10.1186/s13072-018-0199-8) contains supplementary material, which is available to authorized users.

## Background

Assembly of heterochromatin, a dense chromatin structure that represses the expression of embedded genes, is vital for the establishment and maintenance of cell identity. A hallmark of heterochromatin is methylation of histone H3 at Lys-9 (H3K9me), a modification that is conserved from the fission yeast *Schizosaccharomyces pombe* to humans. Studies using *S. pombe* as a model organism have established the concept that the RNA interference (RNAi) pathway contributes to the assembly of heterochromatin (reviewed in [1–4]). In fission yeast, the RNAi pathway is required predominantly at the pericentromeric regions, while the pathway is dispensable for the maintenance of the heterochromatin assembled at the subtelomeric regions and the mating-type locus [5–7]. Notably, defects in the RNAi pathway lead to great loss of H3K9me and derepression of silencing at the pericentromeric regions but not at the subtelomeric regions or the mating-type locus [5–8]. These regional differences in dependence on the RNAi pathway have provided researchers with clues to ascertain whether the factors of interest act specifically in the RNAi pathway or act more generally in the assembly of heterochromatin [9–11].

In the *S. pombe* RNAi pathway, formation of the small interfering RNA (siRNA)-containing effector complex is coupled to heterochromatin assembly [1–4]. siRNA is generated, by the Dicer family endoribonuclease Dcr1, from double-stranded non-coding RNA that is complementary to heterochromatin. The siRNA duplex is loaded onto a non-chromatin-associated complex called Argonaute small interfering RNA chaperone (ARC), which contains the Argonaute family endoribonuclease Ago1. The loading of the siRNA duplex onto the Ago1 subunit requires the two ARC-specific subunits Arb1 and Arb2, which also inhibit the release of the passenger strand [12]. This complex then changes its subunit composition to form a chromatin-associated effector complex called RNA-induced transcriptional silencing (RITS) [12, 13]. The RITS complex is composed of Ago1, now binding single-stranded siRNA as a guide for target recognition, and the two RITS-specific subunits Chp1 and Tas3 [12, 13]. Chp1 uses a chromodomain to recognize H3K9me [14], whereas Tas3 bridges Ago1 and Chp1 [15, 16].

With the ability to interact with both H3K9me and target RNA, RITS plays a central role in the self-enforcing cycle of RNAi-dependent heterochromatin assembly [1–4]. RITS’ function depends on two major interactions. On the one hand, RITS interacts with the RNA-dependent RNA polymerase complex, which synthesizes double-stranded RNA for secondary siRNA generation [17, 18]. On the other hand, RITS interacts (via bridging by the linker protein Stc1 [19]) with the Clr4 histone methyltransferase-containing complex that methylates the H3 histone to create the H3K9me epigenetic marker [20, 21]. Thus, the formation of RITS is crucial for the generation of siRNA and for the assembly of RNAi-dependent heterochromatin.

The formation of small RNA-containing effector complexes is generally assisted by heat-shock molecular chaperones [22–29]. However, the heat-shock molecular chaperones responsible for the RNAi-dependent heterochromatin assembly remain unidentified. The candidates may belong to one or more of the distinct families of heat-shock proteins 40, 70, and 90 (Hsp40, Hsp70, and Hsp90, respectively) [22–24, 30].

Among the three Hsp families, the proteins belonging to the Hsp90 family promote the in vitro formation of small RNA-containing complexes in all species that have been tested [22–24, 29]. Notably, however, Hsp90-family proteins appear to act in species-specific manners. For example, the steps that require ATP hydrolysis by Hsp90-family proteins appear to differ among various species. For instance, Hsp90-mediated ATP hydrolysis is required for siRNA duplex loading in animal cells, but is instead required for passenger strand removal in plant cells [23, 24, 31]. Similarly, the formation of small RNA-containing complexes does not necessarily require Hsp70-family proteins. An Hsp70 protein is essential for complex formation in the fruit fly *Drosophila melanogaster*, but not in the ciliated protozoan *Tetrahymena thermophila* [22, 29]. Therefore, the differences between species should be acknowledged in examining how such chaperones act in RNAi-dependent heterochromatin assembly.

The *S. pombe* genome encodes six Hsp70 proteins. These Hsps show high sequence similarity to their counterparts in the budding yeast *Saccharomyces cerevisiae* (Additional file 1: Fig. S1), where the cellular roles of Hsp70 s have been thoroughly examined [32]. Among the six *S. pombe* Hsp70 proteins, Ssa1 and Ssa2, which show high sequence similarity to each other (identity: 94%), are recognized as nucleocytoplasmic Hsp70 proteins [33]. Ssa1 and Ssa2 also exhibit the strongest sequence similarity to the *D. melanogaster* Hsp70 protein Hsc70-4 (identity: 75% each), which is essential for the formation of a small RNA-containing complex in that organism [22, 30].

The *S. pombe* genome encodes 26 Hsp40 family proteins, all of which harbor a characteristic DnaJ domain (Additional file 1: Fig. S2). These Hsp40 proteins can be divided into three classes: types I, II, and III [34]. Type-I proteins are also found in *S. cerevisiae* (Additional file 1: Fig. S3), and have the same names in the two yeast species [32, 35]. Mdj1 and Scj1 localize in mitochondria and the lumen of the ER, respectively [32]. In contrast, Mas5 (also known as Ydj1 in *S. cerevisiae*) and Xdj1 localize in the cytosol and nucleus [32] and are categorized as nucleocytoplasmic type-I Hsp40 proteins. Among the 26 Hsp40 proteins in *S. pombe*, Mas5 shows the greatest sequence similarity to the *D. melanogaster* protein Droj2 (identity: 41%), a protein that promotes the formation of a small RNA-containing complex in vitro [22, 30].

In the present study, we identified the *S. pombe* molecular chaperone proteins Hsp90 and Mas5 as novel regulators of RNAi-dependent heterochromatin assembly. Mutations in the genes encoding these proteins caused derepression of transcriptional silencing and decreases in H3K9me at the pericentromeric heterochromatin region, while having little effect on the subtelomeric heterochromatin, which can be maintained in the absence of RNAi. Hsp90 and Mas5 were required in vivo for siRNA generation and chromatin localization of Ago1. In addition, the protein level of Tas3 was substantially reduced in the absence of Mas5, suggesting that Mas5 is responsible for the stability of Tas3, and thus of the RITS RNAi effector complex. Therefore, we propose that these molecular chaperones contribute to the assembly of the RNAi-dependent heterochromatin in *S. pombe*.

## Results

### Identification of Hsp90 and Mas5 as silencing factors

In a forward genetic screen for factors that affect pericentromeric silencing [36], we isolated a missense mutation of the *hsp90* gene, which encodes the sole Hsp90-family protein in *S. pombe* (see “Methods” section). In parallel with the genetic screen, we conducted immunoaffinity purification of proteins that interact either with RNA polymerase II or Spt6 and identified an Hsp40-family protein, Mas5, as a silencing factor (see “Methods” section).

**Fig. 1:**
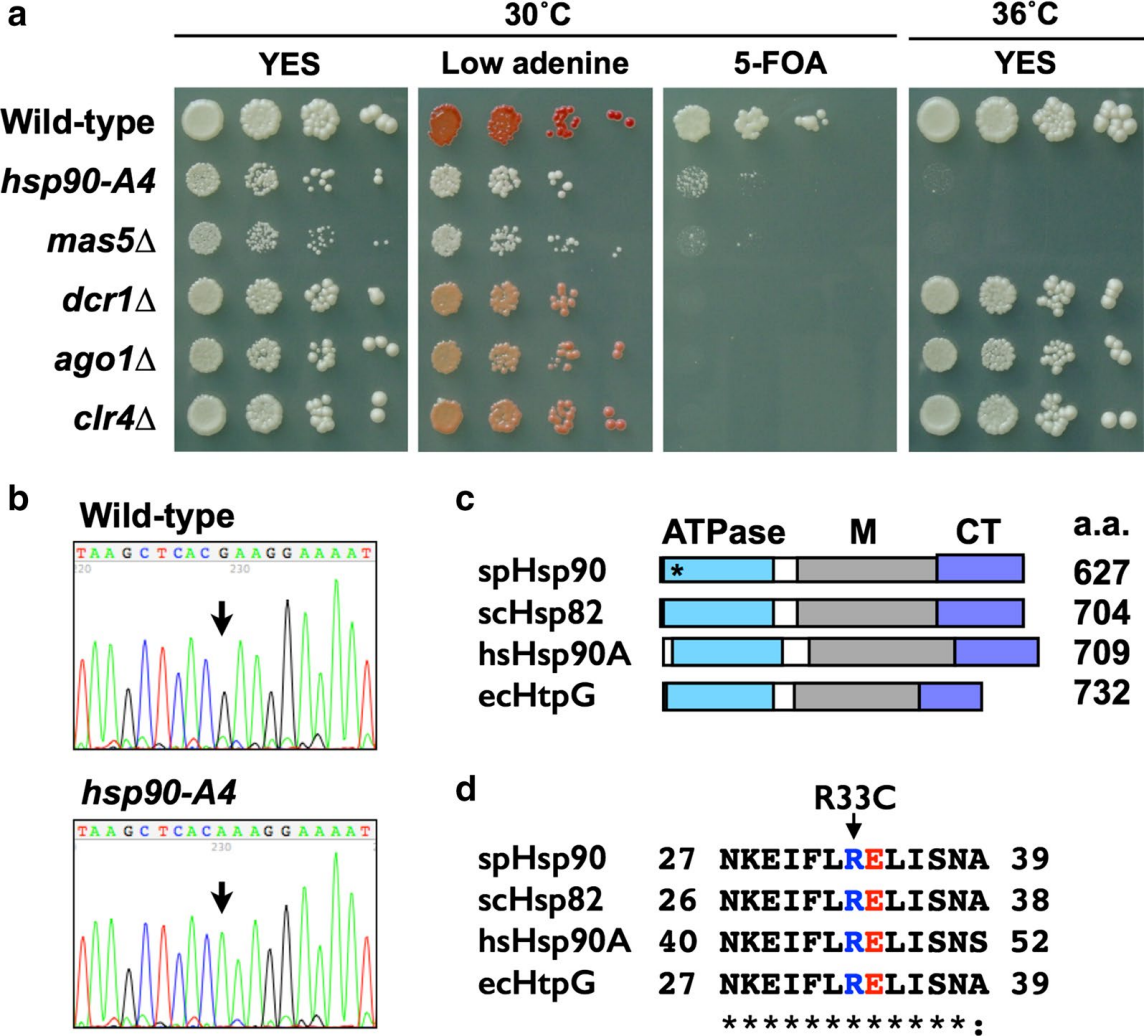
Identification of Hsp90 and Mas5 as silencing factors. **a** Cells were serially diluted, spotted on normal YES plates (YES) and YES containing limited amount of adenine (low adenine) or 0.1% 5-FOA (5-FOA), and incubated at the indicated temperature for 3 days. Marker integration sites are shown in Additional file 1: Figure S4. **b** Results of sequencing of the antisense strand of the *hsp90* gene. Arrows indicate the position of the base substitution. The wild-type cytosine at position 97 (with respect to the ORF start), which is guanine in the antisense strand, was replaced with thymine (adenine in the antisense strand) in the mutant. **c** Domain structure of Hsp90 family proteins. The N-terminal ATPase domains (ATPase), middle domains (M), and C-terminal domains (CT) are indicated. The names of proteins are associated with two-letter abbreviations indicating the species: “sp” for *Schizosaccharomyces pombe* (UniProt id: P41887), “sc” for *Saccharomyces cerevisiae* (P02829), “hs” for *Homo sapience* (P07900), and “ec” for *Escherichia coli* (P0A6Z3). The amino acid length (a.a.) of the proteins is shown. The position of R33C substitution is indicated with an asterisk. **d** Alignment of protein sequences around the R33C mutation. Residue numbers for the first and last amino acid residues for each protein interval are shown. Identical and similar residues are indicated as asterisks and colons, respectively, as seen in a standard ClustalW output. The residues corresponding to the Arg-33 in the fission yeast Hsp90 are colored in blue. The catalytic glutamate residues corresponding to the Glu-33 in budding yeast Hsp82 are colored in red

With the same monitoring system, we evaluated the silencing state of mutant cells by monitoring the expression of *ade6* and *ura4* marker genes embedded in the pericentromere regions [37] (Additional file 1: Fig. S4). In the absence of mutations, this screening strain did not appreciably express *ade6* or *ura4*; thus, cells without a silencing defect formed red colonies on a plate with a limited amount of adenine (due to the accumulation of an intermediate of adenine biosynthesis) and grew healthily on a plate containing 5-fluoroorotic acid (5-FOA) (a pyrimidine precursor analog that is toxic to *ura4*-expressing cells) [37] (Fig. 1a). In contrast, cells bearing mutations in the genes encoding the H3K9 histone methyltransferase Clr4 (*clr4∆*), the Dicer family endoribonuclease Dcr1 (*dcr1∆*), or the Argonaute protein Ago1 (*ago1∆*) formed pink colonies (i.e., decreased accumulation of red pigment) and were sensitive to 5-FOA. This result indicated that heterochromatic silencing at the pericentromere requires each of these factors, in agreement with the literature [1–4]. Importantly, cells harboring a mutation in the genes encoding Hsp90 (*hsp90*-*A4*) or Mas5 (*mas5∆*) formed near-white colonies and exhibited sensitivity to 5-FOA, suggesting that Hsp90 and Mas5 are also required for pericentromeric silencing.

The formation of near-white colonies could be caused by a defect in red pigment formation. However, as *hsp90*-*A4* and *mas5∆* mutant cells not harboring the pericentromeric marker genes formed red colonies (Additional file 1: Fig. S5), Hsp90 and Mas5 appeared not to contribute to pigment formation. In addition, *hsp90*-*A4* and *mas5∆* mutant cells harboring the pericentromeric marker genes grew faster than wild-type cells on Edinburgh minimal medium with supplements (EMMS) lacking adenine (Additional file 1: Fig. S6A), suggesting that the *ade6* marker gene was indeed expressed by these mutations. EMMS medium lacking uracil was not suitable to monitor the expression level of the pericentromeric *ura4* marker gene, because of its leaky repression in the wild-type cells and the slow growth phenotype of the Hsp mutants (Additional file 1: Fig. S6A).

The *hsp90*-*A4* mutant gene had a cytosine-to-thymine base substitution at position 97 (from the start of the ORF) (Fig. 1b), causing a deduced arginine to cysteine substitution at amino acid 33 (R33C). The Arg-33 residue is located in the ATPase domain of Hsp90 and is highly conserved from bacteria to humans (Figs. 1c, d). In *S. cerevisiae*, the corresponding amino acid (Arg-32) has been implicated in the modulation of ATP hydrolysis [38, 39]. Although Arg-32 in the *S. cerevisiae* homolog does not directly contact ATP, the residue forms hydrogen bonds with the adjacent catalytic residue Glu-33 (Glu-34 in the *S. pombe* protein) and with a residue in the Hsp90 middle domain [39]. Through these intramolecular contacts, Arg-32 is thought to be involved in ATP hydrolysis and conformational changes of the Hsp90 protein [38, 39]. Replacement of the *S. pombe* Arg-33 with cysteine is expected to disrupt these intramolecular contacts, implying that the derepression of the pericentromeric silencing in the mutant is caused by a decrease in the ATPase activity of Hsp90.

### Hsp40 protein Xdj1, as well as Hsp70 proteins Ssa1 and Ssa2, is not required for pericentromeric silencing

**Fig. 2:**
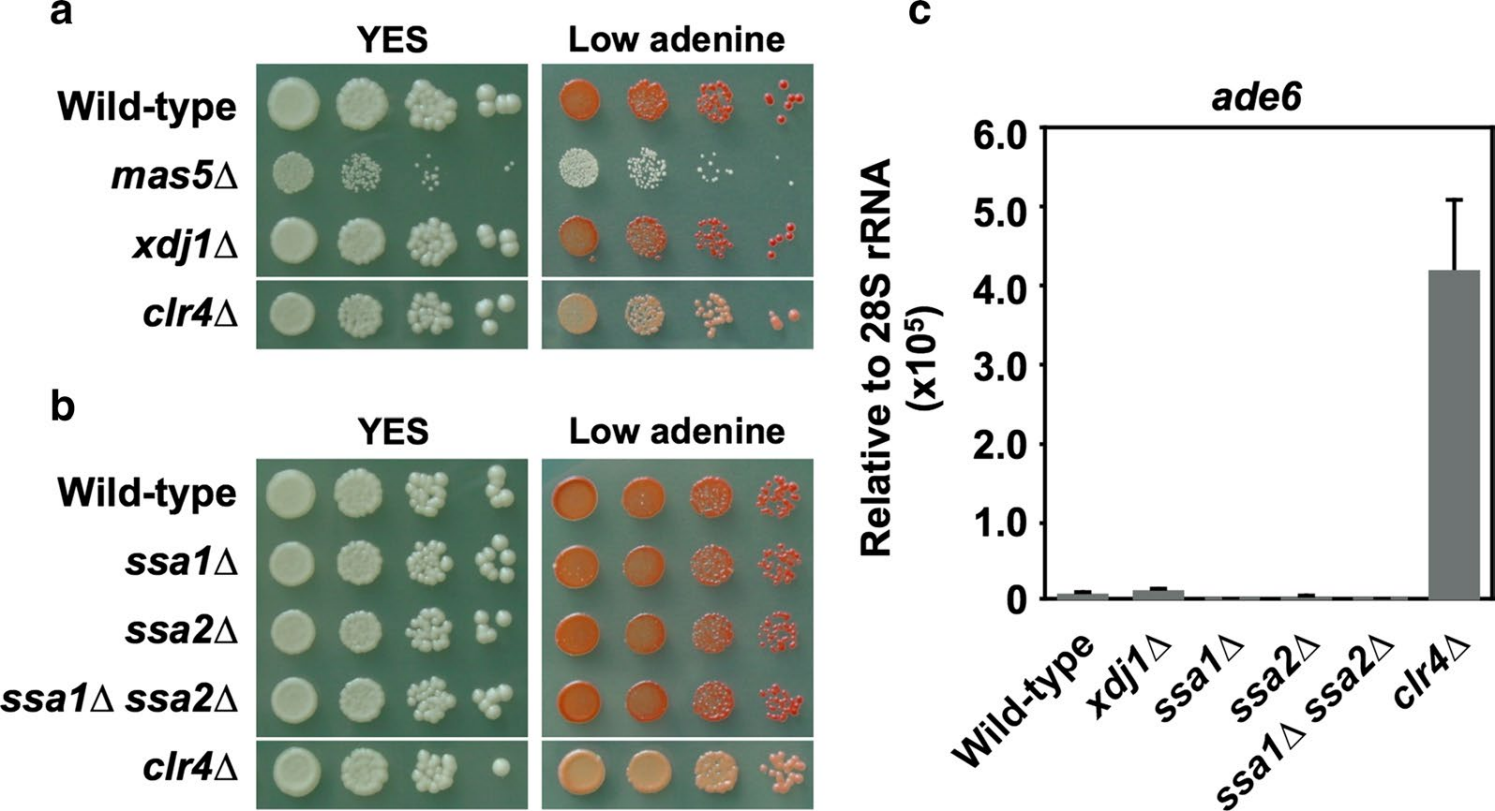
Xdj1, Ssa1, and Ssa2 are dispensable for silencing of the pericentromeric marker gene. **a**, **b** Silencing assay of Hsp40 mutants (**a**) and of Hsp70 mutants (**b**). Cells were serially diluted, spotted on normal YES plates (YES) and YES plates containing limited amounts of adenine (Low adenine), and incubated at 30 °C for 3 days. **c** Strand-specific RT-qPCR for the pericentromeric *ade6* marker gene. Values are presented as means + SD (*n* = 3)

Identification of Hsp90 and Mas5 as silencing factors prompted us to examine whether other molecular chaperones also contribute to pericentromeric silencing. Mas5 is classified as a nucleocytoplasmic type-I Hsp40 protein. There is another protein that falls into this classification: Xdj1 (Additional file 1: Figs. S2 and S3). Therefore, we tested the involvement of Xdj1 in the silencing of the pericentromeric *ade6* marker gene. As shown in Fig. 2a, null mutations in the gene encoding Xdj1 (*xdj1∆*) did not lead to the formation of pink colonies, suggesting that Xdj1 does not have a major role in the silencing.

Mas5 has a DnaJ domain, which has been implicated in the regulation of the ATPase activity of Hsp70 proteins [40, 41]. There are two nucleocytoplasmic Hsp70 proteins in *S. pombe*: Ssa1 and Ssa2 (Additional file 1: Fig. S1). Both Ssa1 and Ssa2 physically interact with Mas5 [33] and (among the *S. pombe* Hsp70 proteins) show the highest sequence similarity to the *D. melanogaster* Hsc70-4. This observation raised the possibility that these two proteins might also act with Mas5 to silence pericentromeric transcription. Nonetheless, as shown in Fig. 2b, single- and double-null mutations in the genes encoding Ssa1 and Ssa2 (*ssa1∆*, *ssa2∆*, and *ssa1∆ ssa2*) did not lead to the formation of pink colonies, indicating that pericentromeric silencing remained intact in the absence of these nucleocytoplasmic Hsp70 proteins.

To confirm that the colony color reflected the transcription of the *ade6* marker gene, we performed strand-specific reverse transcription followed by quantitative polymerase chain reaction (RT-qPCR) analysis (Fig. 2c). Due to the assembly of heterochromatin over the marker gene, the expression level of *ade6* was very low in wild-type cells. In contrast, *ade6* transcription was increased in *clr4∆* cells, in which heterochromatin was not formed. In agreement with the results of the colony color analyses (Fig. 2a, b), null mutations in other genes encoding Hsp40 (*xdj1*) and Hsp70 (*ssa1* and *ssa2*) did not cause substantial increases in the transcript level of the marker gene. Thus, although the nucleocytoplasmic Hsp40 protein Mas5 appears to have a role in pericentromeric silencing, the nucleocytoplasmic Hsp70 proteins Ssa1 and Ssa2 do not appear to be involved in this process.

### Hsp90 and Mas5 are required for RNAi-dependent heterochromatin assembly

**Fig. 3:**
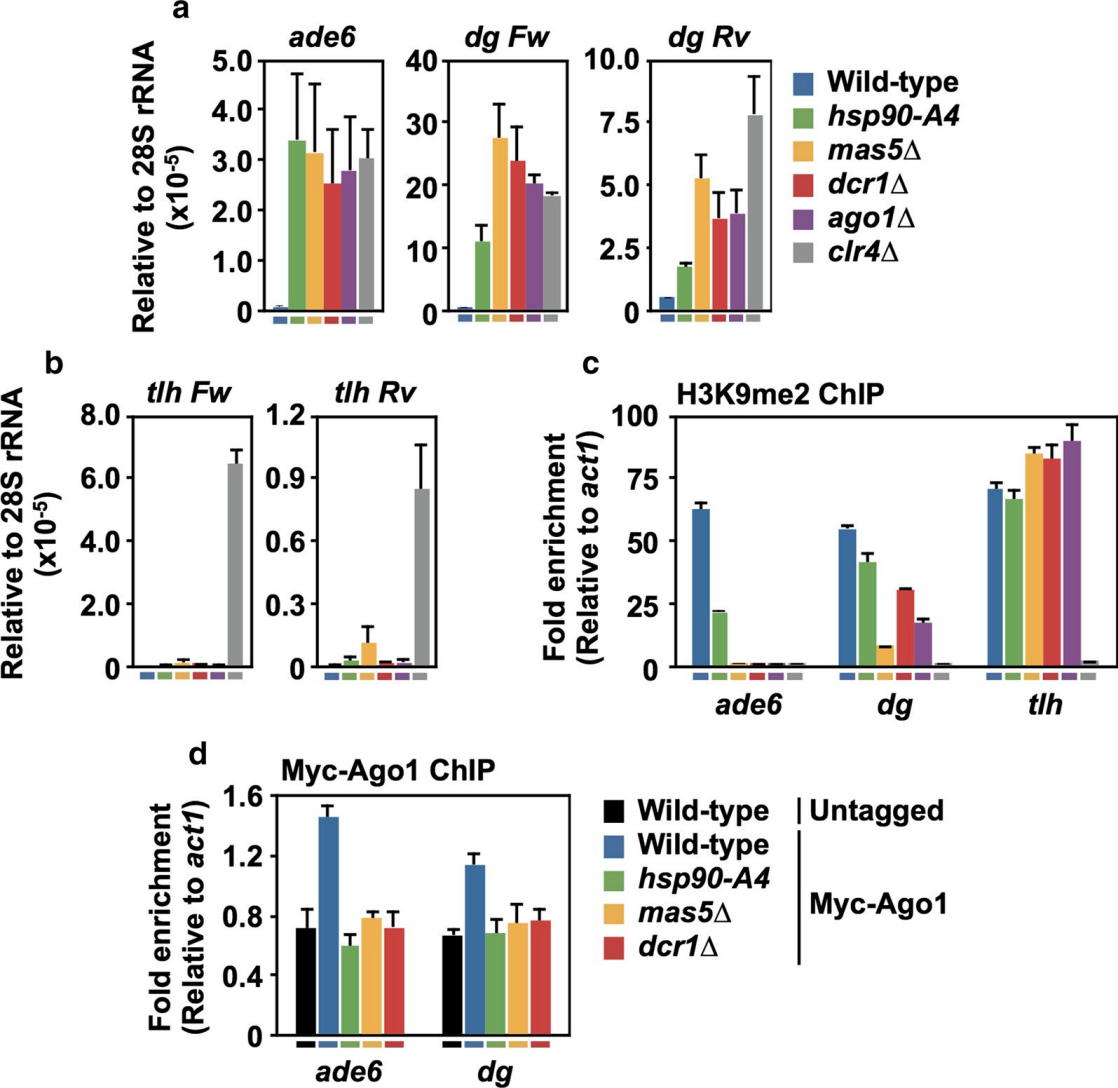
Hsp90 and Mas5 are required for the assembly of heterochromatin at the pericentromere. **a**, **b** Strand-specific RT-qPCR for the pericentromeric transcripts from the *ade6* gene and *dg* repeats (**a**), and for the subtelomeric transcripts from the *tlh* genes (**b**). For the *dg* repeats and *tlh* genes, transcripts matching forward (Fw) and reverse (Rv) strands were analyzed. Values are normalized to that of the sense strand of ribosomal 28S RNA. **c** ChIP-qPCR using an antibody against H3K9me2 for the pericentromeric *ade6* gene, *dg* repeats, and *tlh* genes. Color keys under the graphs in **a**–**c** correspond to the key shown in **a**. The euchromatic gene *act1* was used as an internal control locus. **d** ChIP-qPCR of Myc-Ago1 using an antibody against Myc for the pericentromeric *ade6* gene, *dg* repeats, and *tlh* genes. For all panels, values are presented as means + SD (*n* = 3)

In the *S. pombe* pericentromere, silencing of the inserted marker genes occurs passively, reflecting the chromatin state of neighboring pericentromeric repeat regions. Therefore, we next performed strand-specific RT-qPCR to examine the levels of transcripts from the *ade6* and *ura4* marker genes as well as those from the *dogentai* (*dg*) pericentromeric repeats (Fig. 3a and Additional file 1: Fig. S6B). In wild-type cells, these pericentromeric transcripts accumulated at very low levels, as these regions were silenced by heterochromatin (Additional file 1: Fig. S6C). In contrast, these transcripts accumulated to higher levels in *dcr1∆*, *ago1∆*, and *clr4∆* mutant cells, indicating that silencing was derepressed by these mutations [8, 20]. Importantly, *hsp90*-*A4* and *mas5∆* cells also accumulated the pericentromeric transcripts. These results suggested that Hsp90 and Mas5 are involved in the silencing of both the inserted marker genes and the native pericentromeric repeats.

Derepression of pericentromeric silencing can be caused either by a mutation in the factors that are generally required for heterochromatin assembly, such as those directly involved in H3K9 methylation, or by a mutation in the RNAi factors, which direct H3K9me formation in a locus-specific manner. In the former case, silencing in the subtelomere regions and the mating-type locus, which can be maintained in the absence of RNAi factors [5–7], should also be derepressed; in the latter case, silencing in those non-pericentromeric regions should not be affected. To test the possibility that Hsp90 and Mas5 act as general heterochromatin factors, we examined the expression levels of the subtelomeric *telomere*-*linked helicase* (*tlh*) genes and the *centromere homology* (*cenH*) transcript from the mating-type locus by strand-specific RT-qPCR (Fig. 3b and Additional file 1: Fig. S7). The silencing of the *tlh* genes and of the *cenH* transcript was maintained both in wild-type and RNAi-defective mutants (*dcr1∆* and *ago1∆*), but was derepressed in the presence of *clr4∆*, as reported previously [6, 7, 11]. Notably, silencing of the *tlh* genes was maintained in *hsp90*-*A4* cells and only derepressed by 2–13% in *mas5∆* cells, as compared to the fully depressed state in the *clr4∆* cells. Similarly, silencing of the *cenH* transcript was not markedly affected by the *hsp90*-*A4* or *mas5∆* mutation.

These data suggest that Hsp90 and Mas5 are involved in the RNAi-dependent assembly of heterochromatin. To test this hypothesis, we performed chromatin immunoprecipitation followed by quantitative PCR (ChIP-qPCR) to monitor the level of dimethylation of histone H3 at Lys-9 (H3K9me2) at the *ade6*, *dg*, and *tlh* regions (Fig. 3c). In wild-type cells, the three tested regions exhibited strong enrichment of H3K9me2 compared to that at the euchromatic gene *act1*, which encodes actin. In the absence of the histone methyltransferase Clr4 (*clr4∆*), the H3K9me2 mark was abolished. When the RNAi pathway was defective (*dcr1∆* and *ago1∆*), H3K9me2 was completely abolished at the *ade6* gene, but was only moderately decreased (i.e., 32–55% compared to the wild-type control) at the pericentromeric *dg* repeats and was maintained at the subtelomeric *tlh* genes. These observations are consistent with the results of previous reports [6, 8, 42]. In agreement with the results of RT-qPCR (Fig. 3a, b), *hsp90*-*A4* and *mas5∆* mutations caused reductions in the level of H3K9me2 at the pericentromeric regions but not at the subtelomeres. These results suggest that Hsp90 and Mas5 act like RNAi factors in the assembly of heterochromatin at the pericentromeres.

If Hsp90 and Mas5 are involved in RNAi-dependent heterochromatin assembly, mutations in these proteins may affect chromatin localization of Ago1. Therefore, we constructed mutant strains that also express amino (N)-terminally Myc-tagged Ago1 (Myc-Ago1) from its own promoter [12] and examined the chromatin localization level of Myc-Ago1 by ChIP-qPCR (Fig. 3d). In wild-type cells, Myc-Ago1 was enriched at the *ade6* and *dg* regions when compared to the level at the internal control *act1* gene. This result indicated that the Myc tagging did not perturb the chromatin localization of Ago1. In accordance with a previous report [12], the enrichment of Myc-Ago1 in *dcr1∆* cells was as low as that of the untagged control in the pericentromeric *ade6* and *dg* regions. Similarly, the chromatin localization of Myc-Ago1 in the pericentromeric regions was abrogated in *hsp90*-*A4* and *mas5∆* cells. Therefore, Hsp90 and Mas5 appeared to be required for the localization of Ago1 in the pericentromere.

### Hsp90 and Mas5 are required for the formation of the RNAi effector complex

**Fig. 4:**
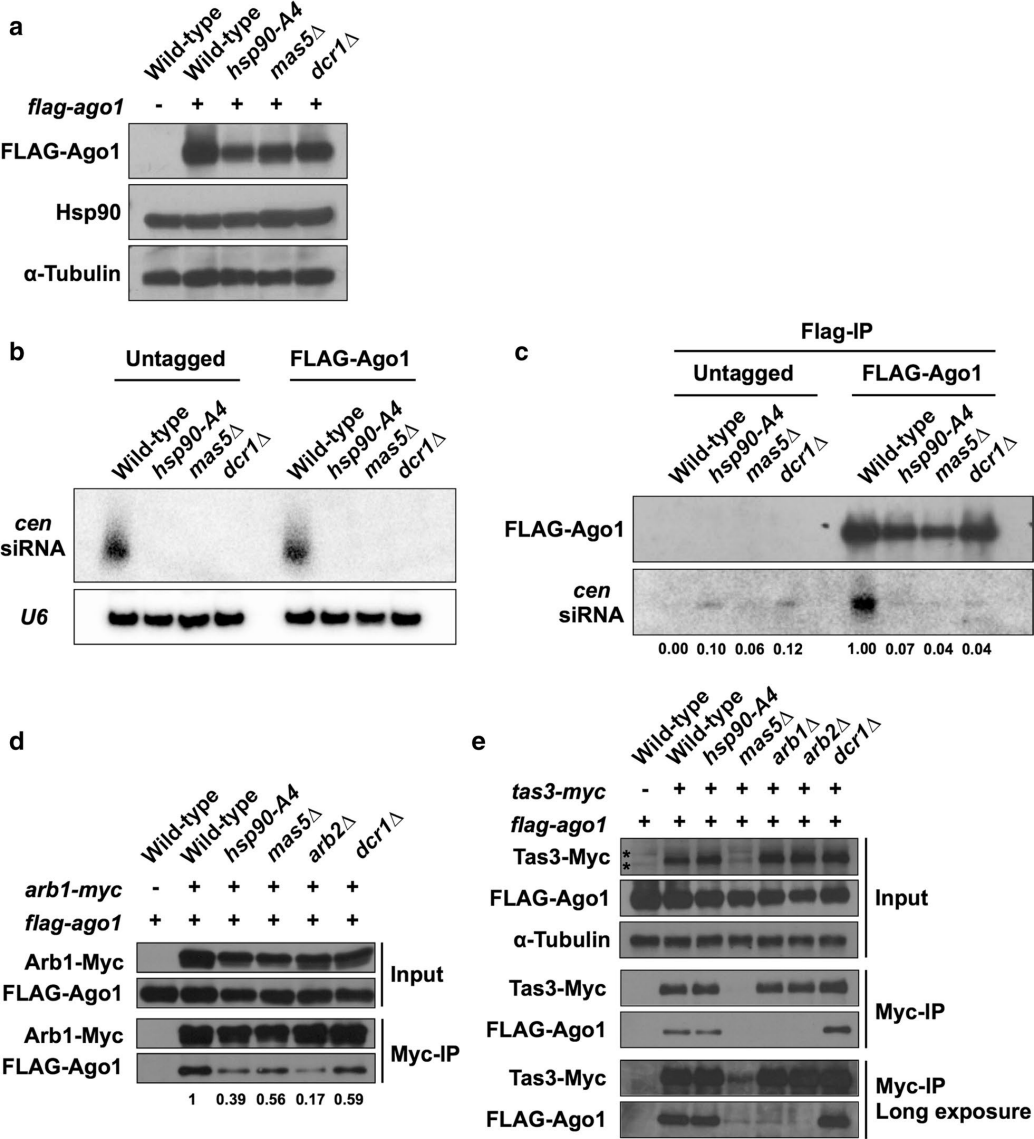
Hsp90 and Mas5 are required for the formation of siRNA-containing Ago1 complexes. **a** Detection of proteins in whole-cell extracts by western blotting. Antibodies against FLAG epitope, Hsp90, and α-tubulin were used. **b** Detection of pericentromeric siRNA. Equal amounts of total RNA extracted from untagged cells or from cells expressing FLAG-Ago1 were loaded into each lane. Pericentromeric siRNA (*cen* siRNA) was detected by northern blotting with specific probes (see “Methods” section). *U6* snRNA was used as a loading control. **c** Detection of pericentromeric siRNA in Ago1 complex. FLAG-Ago1 complex was immunoprecipitated with an antibody against the FLAG epitope. Pericentromeric siRNA extracted from the immunoprecipitates was detected by northern blotting (*cen* siRNA). Signal intensities of the pericentromeric siRNA relative to the wild-type FLAG-Ago1 sample are indicated. Successful immunoprecipitation was validated by means of western blotting with an antibody against the FLAG epitope (FLAG-Ago1). Untagged cells were used as negative controls. **d**, **e** Detection of FLAG-Ago1 in Arb1-Myc (**d**) and Tas3-Myc (**e**) immunoprecipitates. Soluble extracts (Input) and immunoprecipitates (Myc-IP) were separated on SDS-PAGE and detected with antibodies against the Myc and FLAG epitopes; the loading control protein was detected with an antibody against α-tubulin. Asterisks, background signals. Signal intensities of FLAG-Ago1 normalized with those of Arb1-Myc are indicated

As molecular chaperone proteins, Hsp90 and Mas5 may contribute to effector complex formation in the RNAi pathway. To examine this possibility, we first tested whether the protein level of Ago1 is altered in the mutant cells (Fig. 4a). Yeast strains that did (FLAG-Ago1) or did not (untagged) express N-terminally FLAG-tagged Ago1 from its own promoter were used for this analysis [43]. The amount of FLAG-Ago1, Hsp90, and α-tubulin in cell extracts were examined by western blotting. The α-tubulin signals indicated that equal amounts of samples were loaded on the gel. Interestingly, the amount of Hsp90 itself was not altered in *hsp90*-*A4* cells. This observation suggested that the function, rather than the quantity, of the Hsp90 protein is affected by the R33C mutation. As the FLAG-Ago1 signal appeared to be decreased in the mutant cells, we conducted western blotting with twofold serial dilutions (Additional File 1: Fig. S8). The results indicated that the signal intensity of FLAG-Ago1 in *hsp90*-*A4* or *mas5∆* cells was less than a half of that in wild-type cells. These results suggest that Hsp90 and Mas5 are required to maintain the proper amount of Ago1 protein in cells.

Next, we examined the amount of pericentromeric siRNA by northern blotting (Fig. 4b). In total RNA extracts from wild-type cells, we detected 21–24-nt siRNA that were complementary to the pericentromeric repeats; U6 small nuclear RNA was used as a loading control. Comparable amounts of siRNA were detected irrespective of the FLAG tag, indicating that (as described previously [12]) the epitope tagging of Ago1 did not affect siRNA generation. In *dcr1∆* cells, in which siRNA generation should be abolished, the siRNA was not detected, again consistent with previous results [44]. Remarkably, siRNA was undetectable in *hsp90*-*A4* or *mas5∆* cells, suggesting that Hsp90 and Mas5 have major roles in siRNA generation in *S. pombe*.

The above data suggested that Ago1 does not bind appropriate amounts of pericentromeric siRNA in *hsp90*-*A4* and *mas5∆* cells. To confirm this hypothesis, we attempted to detect siRNA in FLAG-Ago1-containing immunoprecipitates by northern blotting (Fig. 4c). Specifically, we subjected extracts from cells expressing FLAG-Ago1 to immunoprecipitation with anti-FLAG antibody. Cells that did not express FLAG-Ago1 were used as negative controls. Successful immunoprecipitation was confirmed by detecting FLAG-Ago1 by means of western blotting (Fig. 4c, FLAG-Ago1). In this analysis, we detected faint background RNA signals of unknown origin even in the immunoprecipitates from untagged strains (Fig. 4c, untagged, *cen* siRNA). However, these RNA signals were very weak (i.e., 6–12% compared to the pericentromeric siRNA signal detected in the immunoprecipitate from wild-type cells expressing FLAG-Ago1) (Fig. 4c, FLAG-Ago1, *cen* siRNA). As expected, the signal intensity of Ago1-bound siRNA from *dcr1∆* cells was approximately 96% weaker than that from wild-type cells. Notably, the Ago1-bound siRNA signals from *hsp90*-*A4* and *mas5∆* cells were comparable to that from *dcr1∆* cells. The amounts of FLAG-Ago1 protein in the immunoprecipitates differed among samples (Fig. 4c, FLAG-Ago1); however, this magnitude of difference did not appear to explain the observed decrease in siRNA. These data suggested that Hsp90 and Mas5 are required for the formation of functional, siRNA-containing effector complex in vivo.

As the loading of siRNA onto Ago1 depends on the formation of the ARC complex [12], we next investigated the interaction between Ago1 and Arb1 in the mutant cells. We constructed strains that co-expressed carboxy (C)-terminally Myc-tagged Arb1 (Arb1-Myc) and FLAG-Ago1 from the respective native promoters. We subjected extracts of the resulting strains to immunoprecipitation of Arb1-Myc, and examined the amounts of Arb1-Myc and FLAG-Ago1 in the immunoprecipitates by western blotting (Fig. 4d). In the soluble extracts (Input) from wild-type strains, comparable amounts of FLAG-Ago1 were detected irrespective of the Myc tagging of Arb1. This result demonstrated that the double tagging did not affect the bulk amount of Ago1 in the wild-type background. In the soluble extracts (Input) from *hsp90*-*A4* and *mas5∆* cells, the signal intensity of Arb1-Myc was slightly lower than that from the wild-type cells. This observation suggests that both Hsp90 and Mas5 are required to maintain Arb1 levels in the cell. It is possible that Hsp90 regulates Arb1 at the RNA level, as the expression of *arb1* mRNA was decreased in *hsp90*-*A4* mutant cells (Additional file 1: Fig. S9). Nonetheless, comparable amounts of Arb1-Myc were detected in the immunoprecipitates (Myc-IP) from Arb1-Myc-expressing cells, permitting further analysis of the interaction between Arb1 and Ago1. Notably, in the wild-type background, FLAG-Ago1 was detected in an Arb1-Myc-dependent manner, indicating that double tagging did not disrupt the interaction between Ago1 and Arb1. In contrast, in the immunoprecipitates from *hsp90*-*A4*, *mas5∆*, and *arb2∆* cells, Arb1-associated Ago1 signals were lower than that in the wild-type sample (Fig. 4d, Myc-IP). These observations suggested that Hsp90 and Mas5 contribute to the formation of the ARC complex in vivo, as previously reported for Arb2 [12].

Next, we examined whether the formation of the RITS complex in vivo is affected by the mutations. We constructed strains that co-express C-terminally Myc-tagged Tas3 (Tas3-Myc) and FLAG-Ago1 from the respective native promoters. Unexpectedly, Tas3-Myc protein was difficult to detect in the *mas5∆* background (Fig. 4e, Input; Additional file 1: Fig. S10). RT-qPCR analysis showed that *tas3* mRNA accumulated to higher (not lower) levels in the *mas5∆* mutant (compared to the wild-type background) (Additional file 1: Fig. S9). This observation suggested that Mas5 is required to stabilize the level of Tas3 protein in *S. pombe* cells.

When wild-type extracts were subjected to immunoprecipitation with anti-Myc antibody, FLAG-Ago1 was detected in the immunoprecipitates in a Tas3-Myc-dependent manner (Fig. 4e, Myc-IP), indicating that Tas3-Myc formed a complex with FLAG-Ago1 in vivo. FLAG-Ago1 also was detected in the immunoprecipitates from *dcr1∆* cells, as reported previously [45]. In *arb1∆* and *arb2∆* cells, the amount of FLAG-Ago1 interacting with Tas3-Myc was decreased to background levels (Fig. 4d, Myc-IP long exposure), indicating that the ARC subunits Arb1 and Arb2 are required for the formation of RITS, consistent with the previous report [45]. However, comparable amounts of FLAG-Ago1 were detected in the Tas3-Myc immunoprecipitates in wild-type and *hsp90*-*A4* cells, suggesting that this *hsp90* mutation does not impair the interaction between Ago1 and Tas3.

## Discussion

**Fig. 5:**
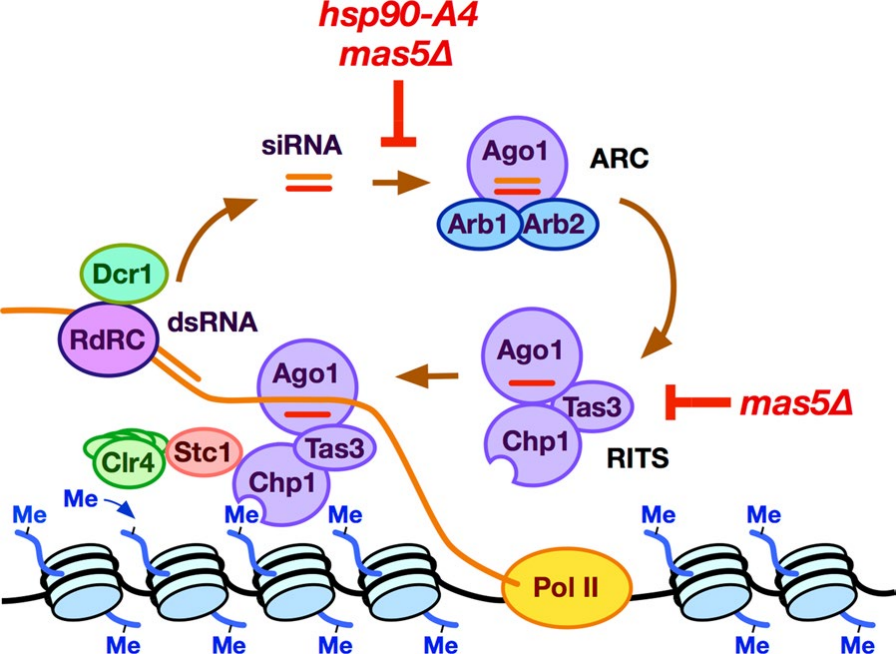
Model. Formation of the siRNA-containing complexes ARC and RITS are key steps in the assembly of RNAi-dependent heterochromatin at the pericentromere. In ARC and RITS complexes, the Argonaute protein Ago1 binds double-stranded and single-stranded siRNA, respectively. The subunits of the non-chromatin-associated ARC complex are responsible for the loading of siRNA onto Ago1, which is then incorporated into the RITS complex. RITS acts in the center of the self-enforcing loop of RNAi-dependent heterochromatin assembly. RITS recruits the RNA-dependent RNA polymerase complex (RDRC) for siRNA generation, while also recruiting (via Stc1) the Clr4-containing methyltransferase complex that methylates H3K9. The generation of siRNA and loading of siRNA onto Ago1 in vivo require Hsp90 and Mas5. Mas5 also maintains the protein level of the RITS subunit Tas3. Thus, Hsp90 and Mas5 are required for RNAi-dependent heterochromatin assembly

In this study, we demonstrated that the *S. pombe* molecular chaperones Hsp90 and Mas5 are required for the silencing, heterochromatin assembly, and chromatin localization of Ago1 in the pericentromere (Figs. 1 and 3). In contrast, the heterochromatin assembled at the subtelomeric regions and mating-type locus, which can be maintained in the absence of RNAi, was not strongly affected by the mutations in Hsp90 or Mas5 (Fig. 3 and Additional file 1: Fig. S7). We also showed that the in vivo generation of siRNA complementary to the pericentromeric repeats required these chaperones (Fig. 4b, c). Furthermore, we showed that Mas5 contributes to maintenance of protein levels of Tas3 in the cells (Fig. 4e). Together, these results indicated that Hsp90 and Mas5 are involved in RNAi-dependent heterochromatin assembly in *S. pombe* (Fig. 5).

Hsp90 has been shown to promote the formation of an RNAi effector complex in plants and animals [22–24, 30]. However, previous studies on the relationship between Hsp90 and effector complexes were based mainly on in vitro experiments; few of these studies examined the in vivo effects of Hsp90 inhibition on the chromatin state. Notably, previous works did not examine whether Hsp90 is required for RNAi-dependent heterochromatin assembly. In this regard, the discovery of Hsp90 as a silencing factor in *S. pombe* may be an important step for understanding how this chaperone contributes to the epigenetic regulation of chromatin formation.

We observed that some H3K9me2 remained at the pericentromeric *ade6* marker gene in *hsp90*-*A4* cells, while H3K9me2 levels were decreased to background levels in *mas5∆* cells (Fig. 3c). Given this result, we cannot confidently state that Hsp90 is essential for RNAi-dependent heterochromatin assembly. The residual H3K9me2 may reflect residual activity of Hsp90 and residual siRNA, which would be technically difficult to detect as a positive signal by Northern blotting, in the *hsp90*-*A4* cells. Alternatively, Hsp90 may be important, but not essential, for RNAi-dependent heterochromatin assembly. The *hsp90* gene is essential for growth, precluding silencing analysis in a gene deletion mutant. The development of specific genetic tools may be necessary to determine whether Hsp90 is essential for RNAi-dependent heterochromatin assembly in vivo.

Hsp40 proteins act as modulators of Hsp70 proteins [32, 46, 47]. In *D. melanogaster*, the Hsp70 protein Hsc70-4 physically interacts with Argonaute proteins and is essential for the formation of RNAi effector complexes. The *D. melanogaster* Hsp40 protein Droj2 also is associated with Argonaute proteins [22, 30]. Droj2 protein is not essential for the effector complex formation but has been shown to promote the effector complex formation in vitro [30]. Droj2 is categorized as a nucleocytoplasmic type-I Hsp40 and exhibits higher sequence identity to the *S. pombe* Mas5 (41%) than to the other *S. pombe* paralog Xdj1 (29%). Therefore, it is possible that Mas5, as a conserved Hsp40 protein, promotes the formation of the RNAi effector complex in the *S. pombe* cells.

Although we identified a nucleocytoplasmic type-I Hsp40 (Mas5) as a silencing factor that is essential for RNAi-dependent heterochromatin assembly, we demonstrated that the double-null mutation in the genes encoding the nucleocytoplasmic Hsp70 proteins Ssa1 and Ssa2 does not cause a detectable defect in pericentromeric silencing in fission yeast (Fig. 2). Ssa1 and Ssa2 each show 75% identity to *D. melanogaster* Hsc70-4. Involvement of the remaining four *S. pombe* Hsp70 proteins in silencing is difficult to imagine, given that these fission yeast paralogs exhibit restricted intracellular locations. Interestingly, in the ciliated protozoan *T. thermophila*, inhibition of Hsp70 does not impair the formation of the effector complex in vitro [29]. Therefore, it is possible that nucleocytoplasmic Hsp70 is dispensable for RNAi-dependent heterochromatin assembly in *S. pombe*. In such cases, Mas5 may be acting in an Hsp70-independent manner: DnaJ domain-independent functions of Hsp40 proteins have been proposed in many studies, as reviewed in references [46, 47].

Despite several attempts to detect physical interactions between the *S. pombe* heat-shock molecular chaperones and Ago1 using co-immunoprecipitation from cell extracts, we were unable to detect a positive signal stronger than background level (data not shown). This suggests that the assumed physical interaction is transient, or that the involvement of these molecular chaperones in the formation of the siRNA-containing effector complexes is indirect at the molecular level. As the *S. pombe* RNAi pathway forms a self-reinforcing loop that is required for and is coupled to the assembly of the pericentromeric heterochromatin, a defect in any step in the loop may result in essentially the same result: loss of siRNA generation [17]. Thus, we cannot rule out the possibility that the formation of the effector complex is inhibited in vivo because the mutations in Hsp90 and Mas5 inhibit other steps in the RNAi pathway. Notably, the decrease in Tas3 protein levels observed in *mas5∆* cells, (Fig. 4e and Additional file 1: Fig. S10) may eliminate siRNA generation. Thus, Mas5 may contribute to RNAi-dependent heterochromatin formation by maintaining the amount of functional Tas3. Note that Mas5 has been affinity-captured by Stc1 [19], suggesting a contribution of Mas5 in RNAi-dependent heterochromatin formation through this interaction. The *hsp90*-*A4* and *mas5∆* mutants grew much more slowly than the wild-type control, while also exhibiting temperature-sensitive growth; these phenotypes were not seen in the other silencing mutants evaluated here (Fig. 1a). Thus, Hsp90 and Mas5 appear to have roles beyond the RNAi pathway, affecting cell growth and tolerance to heat stress.

Colony colors of canonical heterochromatin mutants (i.e., *clr4∆* and *dcr1∆*) harboring the *ade6*^+^ marker gene in the pericentromere (*otr1R(SphI)::ade6*^+^) are often described as “white” or “light pink” in the literature [19, 48, 49]. This is true when the auxotrophic missense allele *ade6*-*m210*, which can be interallelically complemented by another auxotrophic allele *ade6*-*m216* [50], is located in the endogenous *ade6* locus on Chromosome III (Additional file 1: Fig. S11). However, when the deletion allele *ade6*-*DN/N* is used instead of *ade6*-*m210*, the canonical heterochromatin mutants form apparently darker “pink” colonies [36] (Additional file 1: Fig. S11). Similarly, colony colors of trichostatin A-treated cells are dependent on the endogenous *ade6* alleles [37]. Thus, the *ade6*-*DN/N*-driven enhanced pigmentation may help us visually examine the degree of silencing defects [36].

We noted that *hsp90*-*A4* and *mas5∆* cells formed much “brighter” colonies and grew healthier than *clr4∆* or *dcr1∆* cells on the adenine-limiting (Fig. 1a) and adenine-lacking (Additional file 1: Fig. S6A) plates, respectively. The colony brightness phenotype of *hsp90*-*A4* and *mas5∆* mutants in the *ade6*-*DN/N* background was characteristic of this class of mutants, permitting them to be readily distinguished from canonical silencing mutants such as *clr4∆* [36]. While other bright colony-forming silencing mutants have been isolated in similar forward genetic screens, all of those mutants exhibited alterations in RNA polymerase II-driven transcription [9, 11, 36]. Although the mechanism that causes this brightness is not yet clear, studying the defect caused by these mutants may lead to an understanding of a yet-unknown regulatory layer of epigenetic silencing. In this regard, colony colors of heterochromatin mutants that have been studied in the *ade6*-*m210* background [19, 48, 49, 51] might be worth being examined in the *ade6*-*DN/N* background to visually classify the mutations.

## Conclusions

Based on the results presented in this study, we propose that molecular chaperones Hsp90 and Mas5 are required for RNAi-dependent heterochromatin assembly in *S. pombe*. Although the underlying molecular mechanism remains to be elucidated, mutations in the genes encoding these chaperones greatly decreased the levels of pericentromeric siRNA and H3K9me2 in vivo. Our results suggest that inhibition of the counterparts of these chaperones in other species may have similar destructive effects on chromatin regulation.

## Methods

*Genetic manipulations* The *S. pombe* strains and primers for genetic manipulations used in this study are listed in Additional file 2: Tables S1 and S2, respectively. General yeast manipulation methods and culture conditions were as documented elsewhere [36, 52]. For N-terminal tagging of Ago1, p3FLAGago1N-natMX4 or p3MYCago1N-natMX4 was integrated into the *ago1* locus. To construct these plasmids, 3xFLAG or 3xMyc (respectively) epitope-encoding sequences, including an *Nde*I linker, were inserted between the promoter (extending from nt -259 to the ORF start codon) and the sequences encoding the N-terminus (from the second codon to nt 733 of the ORF) of the *ago1*^+^ gene in the *natMX* gene plasmid. The respective plasmids were digested with *Xho*I, purified by ethanol precipitation, and introduced into host strains using a yeast transformation kit for *S. pombe* (Wako Pure Chemical Industries) according to the manufacturer’s instructions. clonNAT-resistant clones were selected on YES plates containing 100 mg/L clonNAT (Werner BioAgents). All of the tagged sequences were subjected to DNA sequencing to confirm that no additional mutation had been introduced during construction.

*Chromatin immunoprecipitation* Chromatin immunoprecipitation and subsequent qPCR were performed as described elsewhere [36]. The primary antibody used for immunoprecipitation of H3K9me2, the secondary antibody-conjugated magnetic beads, and the primers used for qPCR were as described in the previous study [36]. Cells growing logarithmically in YES medium at 30 °C were used for the analyses. Cell density in each culture was measured with a particle counter (CDA-500, Sysmex) according to the manufacturer’s instructions. To avoid inaccurate measurements caused by cell flocculation, cells were diluted and briefly sonicated by directly immersing the cuvettes in an ultrasonic cleaner bath (Branson 5510) prior to counting.

*RNA preparation, RT-qPCR, and northern blotting* Methods for total RNA extraction, RT-qPCR, and northern blotting were as documented elsewhere [11, 36, 44]. For the northern blotting of pericentromeric siRNA, we used oligonucleotide DNA probes that are complementary to the sequenced siRNAs named “A” to “L” [44, 53]. The siRNA named “D” (5′-UGGAUUAAGGAGAAGCGGUA-3′) [53] was omitted for northern detection, because the probe complementary to this sequence tends to detect unwanted background RNA. For northern blotting of RNA in FLAG-Ago1 immunoprecipitates, RNA was prepared as follows. Extracts of cells growing logarithmically in YES at 30 °C were obtained as described in the immunoprecipitation and western blotting section. For each biological sample, 4 × 10^8^ cells (two tubes for one biological sample) were used for the analysis. The cell extracts from the two tubes were combined, and 440 µL of IP buffer (50 mM HEPES–KOH, pH 7.5, 140 mM NaCl, 1 mM EDTA, 1% Triton X-100, and 0.1% Na-deoxycholate) containing protease inhibitors (P08215, Sigma-Aldrich) was added to obtain 880 µL of whole-cell extract for each biological sample. An aliquot (200 µL) of secondary antibody-conjugated magnetic beads was prepared as described in the immunoprecipitation and western blotting section. After centrifuging the pooled cell extract at 20,000×*g* at 4 °C for 15 min, an aliquot (850 µL) of the resulting supernatant was incubated with the magnetic beads for 2 h at 4 °C. Beads were washed twice with 500 µL per wash of IP buffer containing protease inhibitors and resuspended in 500 µL of IP buffer containing protease inhibitors. Of the 500 µL of immunoprecipitate, one 100 µL aliquot was stored for evaluation by western blotting. For the remaining 400 µL, the supernatant was removed and the beads were suspended in 250 µL of AE buffer (50 mM sodium acetate pH 5.2, 10 mM EDTA). After mixing with 250 µL of citrate-saturated phenol, samples were frozen at − 80 °C and thawed at 65 °C. After centrifugation at 20,000×*g* at 25 °C for 5 min, the aqueous layer was mixed with 250 µL of phenol/chloroform/isoamyl alcohol (25:24:1). After centrifugation at 20,000×*g* at 25 °C for 5 min, the resulting aqueous layer was mixed with 25 µL of 3 M sodium acetate, pH 5.2, 625 µL of ethanol, and 2 µL of Ethachinmate (312-01791, Nippon Gene) and centrifuged at 20,000×*g* at 4 °C for 20 min. The pellet was rinsed with 500 µL of 80% ethanol and resuspended in 20 µL of DEPC-treated water. An aliquot (10 µL) of the RNA sample was used for the northern analysis.

*Immunoprecipitation and western blotting* Cells (2 × 10^8^) growing logarithmically in YES at 30 °C were washed with 10 mL of distilled water and with 1 mL of IP buffer. Cell pellets were resuspended in 220 µL of IP buffer containing protease inhibitor cocktail. Cells were disrupted in a Multi-Beads Shocker (MB400U, Yasui Kikai). IP buffer (400 µL) containing protease inhibitors was added to the cell extract, and the mixture was then centrifuged at 20,000×*g* at 4 °C for 15 min to obtain the supernatant as input extract. An aliquot (200 µL) of M-280 anti-mouse sheep antibody-conjugated magnetic beads (112-02, Thermo Fisher Scientific) was washed twice with IP buffer, incubated with 3 µL of mouse monoclonal anti-FLAG antibody (M2, Sigma-Aldrich) at 4 °C for 2 h, and washed three times with IP buffer. Beads were resuspended in 500 µL of the input extract, incubated at 4 °C for 2 h, washed three times with 500 µL per wash of IP buffer containing protease inhibitors, and resuspended in 50 µL of SDS sample buffer. Western blotting and detection of epitope-tagged proteins were performed as described previously [52]. Mouse monoclonal anti-Myc antibody 9E10 (1:2000), anti-G196 ascites (1:2000), anti-α-tubulin antibody DM1A (1:2000), and anti-FLAG antibody M2 (1:2000) were used as primary antibodies. HRP-conjugated goat anti-mouse antibody (1:5000, Rockland Immunochemicals) was used as secondary antibody.

*Isolation of hsp90-A4 silencing mutant* In a genetic screen described previously [36], six mutants that exhibited a defect in pericentromeric silencing were isolated; characterization of three of these mutants was reported elsewhere [11, 36]. For the present work, we characterized one of the remaining three mutants, *A4*, which was named after its original mutant pool designation (“A”). Sequence analysis of the whole genome of an *A4* strain was performed as described previously [36]. Five missense mutations were found in the protein-coding genes of the *A4* genome. Genetic analysis of the *A4* strain revealed that a mutation in the *hsp90* gene, causing the R33C mutation, could not be genetically separated from the *A4* phenotype: all of the tested *A4* progeny with the silencing phenotype possessed the mutation (*n* = 9). Reintroduction of the mutation into the wild-type genome via a selective marker (*kanMX::hsp90*-*A4*) as described previously [36] yielded the same phenotype as that observed in the original *A4* mutant (Additional file 1: Fig. S12). Therefore, we named the mutant allele *hsp90*-*A4*.

*Identification of Mas5 as a silencing factor* In order to identify candidate proteins that might act as silencing factors, we screened for proteins that interacted with either RNA polymerase II or Spt6, an RNA polymerase II-associated histone chaperone, both of which are involved in heterochromatic silencing [36, 54, 55]. G196-tagged protein complexes were immunoprecipitated from four strains [52]: a wild-type strain not expressing a G196-tagged protein (HKM-1100); a wild-type strain expressing C-terminally G196-tagged Spt6 from its own promoter (HKM-2064); a *iws1∆* strain expressing the G196-tagged Spt6 (HKM-2066); and a wild-type strain expressing C-terminally G196-tagged Rpb3, an RNA polymerase II subunit, from its own promoter (HKM-2061).

For preparing anti-G196 antibody-conjugated Sepharose beads, 500 µL of Protein G-Sepharose (4 Fast Flow, GE Healthcare) was washed three times with 10 mL per wash of 20 mM Tris–HCl, pH 8.0. The beads were resuspended in 10 mL of 20 mM Tris–HCl, pH 8.0, mixed with 2 mL of anti-G196 murine ascites, and incubated overnight under rotation at 4 °C. After removal of the supernatant, the beads were washed three times with 10 mL per wash of borate buffer (0.2 M sodium borate, pH 9.0). The beads were resuspended in borate buffer containing 20 mM of dimethyl pimelimidate dihydrochloride (D8388, Sigma-Aldrich), rotated for 30 min at room temperature, and washed twice with 10 mL per wash of 0.2 M ethanolamine, pH 8.0. Beads then were resuspended in 10 mL of 0.2 M ethanolamine, pH 8.0; rotated for 2 h at room temperature; washed twice with 10 mL per wash of phosphate-buffered saline (PBS); washed once with 10 mL of 100 mM glycine–HCl, pH 2.5; washed twice with 0.2 M Tris–HCl, pH 8.0; washed twice with 10 mL per wash of IP buffer containing 0.05% sodium azide; and finally suspended in 500 µL of IP buffer containing 0.05% sodium azide. The resulting antibody-conjugated Sepharose beads were stored at 4 °C.

Extracts of cells growing logarithmically in YES at 30 °C were obtained as described in the immunoprecipitation and western blotting section. For each biological sample, 1 × 10^9^ cells (five tubes for one biological sample) were used for the analysis. Cell extracts from two tubes were combined and 1.3 mL of IP buffer containing protease inhibitors was added to obtain 2.4 mL of whole-cell extract for each biological sample. Insoluble debris was removed by centrifuging the extract three times at 20,000×*g* at 4 °C for 15 min. An aliquot (30 µL) of the anti-G196 antibody-conjugated Sepharose beads (prepared as described above) were washed with 1 mL of IP buffer. The beads were suspended in a 1-mL aliquot of the soluble extract and incubated for 3 h at 4 °C under rotation. After removal of the supernatant, the beads were suspended in another 1-mL aliquot of the soluble extract and incubated for 3 h at 4 °C under rotation. The beads were washed four times with IP buffer containing protease inhibitors and finally suspended in 10 mL of sample buffer. The precipitates were separated on a 12% SDS-PAGE gel, silver-stained (Additional file 1: Fig. S13), and subjected to mass spectrometric analysis as described previously [56]. Proteins identified with over 95% probability were assigned using the scaffold3 software ver. 3.5.1 (Proteome Software, Inc.). Isolation of transcription-related proteins in an Spt6- or Rpb3-dependent manner was confirmed by successful, selective co-immunoprecipitation (Additional file 2: Table S3). Non-essential genes corresponding to ten of these proteins were (individually) genetically deleted and evaluated for contribution to the silencing of the *ade6* marker gene inserted in the pericentromere by observing the colony color of deletants growing on YES medium that contained limiting amounts of adenine (Additional file 2: Table S4). In this screen, Mas5 was identified as a potential silencing regulator.

*Drawing of phylogenetic trees and of schematic representations of homologous proteins* Amino acid sequences and Gene3D domain information for Hsp40 and Hsp70 proteins were obtained using the BioMart tool of Ensemble (Release 35). The dataset for the *S. pombe* proteins was ASM294v2; *S. cerevisiae*, R64-1-1; and *D. melanogaster*, BDGP6. Phylogenetic trees in Additional file 1: Figs. S1 and S3 were drawn with the web software Phylogeny.fr [57]. Order of the proteins was manually changed on the web site. Colors for protein names were changed (using the vector design software Graphic ver. 3.0.1 (Autodesk, Inc.)) by processing the scalable vector graphics images that were downloaded from the Phylogeny.fr web site. Schematic representations of proteins in Additional file 1: Figs. S1–S3 were drawn with the generic graphic functions “plot” and “polygon” of the R statistical environment. Localization signals and transmembrane helices were predicted with the web software TargetP, ver. 1.1 [58], and TMHMM, ver. 2.0 [59], respectively.

## Additional files

**Additional file 1: Figure S1.** Fission yeast Hsp70 proteins and their homologs. **(A)** Phylogenic tree of Hsp70 proteins. Scale-bar unit indicates the number of amino acid substitutions per site. Names of proteins are associated with two-letter abbreviations and color-coded to indicate the species: “sp” for *Schizosaccharomyces pombe* (red), “sc” for *Saccharomyces cerevisiae* (black), and “dm” for *Drosophila melanogaster* (blue). **(B)** Domain structure of Hsp70 proteins. Protein names are depicted as in (A) and their amino acid lengths are shown. Protein domains are drawn as boxes according to the CATH-Gene3D classification. The N-terminal ATPase domains (ATPase) are composed of three internal domains that belong to the CATH superfamilies 3.30.420.40, 3.30.30.30, and 3.90.640.10. Substrate-binding domains (SB) belong to the 2.60.34.10 superfamily. The C-terminal lid domains (Lid) belong to the 1.20.1270.10 superfamily. Predicted localization signals for mitochondria (TargetP-M) and for the endoplasmic reticulum or beyond (TargetP-S) are shown as shaded boxes. Locations of the proteins in the cell are indicated. For clarity, *D. melanogaster* Hsp70 proteins other than Hsc70-4 are omitted. **Figure S2.** Hsp40 family proteins in fission yeast. Domain structure of all 26 fission yeast Hsp40 proteins are shown. Protein names and amino acid lengths are indicated. Protein domains are drawn as boxes according to the CATH-Gene3D, Pfam, and Prosite classifications. Predicted localization signals are shown as in Additional file 1: Figure S1. Predicted trans-membrane helix regions (TMhelix) are indicated as gray boxes. The DnaJ domains belong to the CATH superfamily 1.10.287.110. Type-I Hsp40 proteins are characterized by C-terminal (purple) and central (pink) domains that belong to the CATH superfamilies 2.60.260.20 and 2.10.230.10, respectively. The type-II Hsp40 protein Psi1 also contains the C-terminal domain but lacks the central domain. The other proteins are classified as the type-III Hsp40 proteins. **Figure S3.** Fission yeast type-I and -II Hsp40 proteins and their homologs. **(A)** Phylogenic tree of Hsp40 proteins. Scale-bar unit indicates the number of amino acid substitutions per site. Names of proteins are depicted as in Additional file 1: Figure S1. **(B)** Domain structure of Hsp40 proteins. Protein names are depicted as in (A) and amino acid lengths are shown. Protein domains and predicted localization signals are drawn as in Additional file 1: Figure S2. Locations of proteins in the cell are indicated. For clarity, *D. melanogaster* Hsp40 proteins other than Droj2 are omitted. **Figure S4.** Schematic representation of marker integration sites. The *ade6*^+^ and *ura4*^+^ marker genes were located in the *Sph*I site of *otr1R* (*otr1R(SphI)::ade6*^+^) and in the *Nco*I site of *imr1L* (*imr1L(NcoI)::ura4*^+^), respectively. **Figure S5.** Hsp90 and Mas5 are dispensable for red pigment formation. Cells were spotted on normal YES plates (YES) and YES containing limited amount of adenine (Low adenine), and incubated at 30°C for six days. **Figure S6.** Derepression of marker genes inserted in the pericentromere. **(A)** Cells were serially diluted, spotted on normal EMMS plates and EMMS lacking adenine (EMMS - adenine) or uracil (EMMS - uracil), and incubated at 30°C for 6 days. **(B)** Strand-specific RT-qPCR for the *ura4* marker gene. Values are normalized to that of the sense strand of ribosomal 28S RNA, and are presented as means + SD (*n* = 3). **(C)** Strand-specific RT-qPCR for the *ade6* and *ura4* genes located in the endogenous loci (*ade6*^+^ and *ura4*^+^) and in the pericentromere (*otr1R::ade6*^+^ and *imr1L::ura4*^+^). Values are normalized to that of the sense strand of ribosomal 28S RNA, and are presented as means + SD (*n* = 3). **P < 0.01 (Student’s t-test). **Figure S7.** Hsp90 and Mas5 are dispensable for the silencing at the mating-type locus. Strand-specific RT-qPCR for the *cenH* transcript from the mating-type locus. Values are normalized to that of the sense strand of ribosomal 28S RNA, and are presented as means + SD (*n* = 6). **P < 0.01 (Student’s t-test). **Figure S8.** Reduction of Ago1 protein level in *hsp90-A4* and *mas5∆* cells. Two-fold serially diluted whole-cell extracts were separated by SDS-PAGE followed by western blotting and Coomassie brilliant blue staining. The membrane for detecting FLAG-Ago1 with an antibody against FLAG epitope was reprobed with an antibody against α-tubulin. **Figure S9.** mRNA expression levels of *arb1* and *tas3* genes. Strand-specific RT-qPCR for the *arb1* and *tas3* genes. Values are normalized to that of the sense strand of ribosomal 28S RNA, and are presented as means + SD (*n* = 6). *P < 0.05, **P < 0.01 (Student’s t-test). **Figure S10.** Protein expression level of Tas3. Detection of proteins in whole-cell extracts by western blotting. The membrane for detecting Tas3-Myc with an antibody against Myc epitope was reprobed with an antibody against α-tubulin to confirm that equal amounts of samples were loaded. For the *mas5* deletion, four independently constructed clones were tested. **Figure S11.** Colony colors of *otr1R::ade6*^+^ strains are dependent on the *ade6* alleles in the endogenous *ade6* locus. Cells were streaked on normal YES plates (YES) and YES containing limited amount of adenine (Low adenine), and incubated at 30°C for four days. Strains with *otr1R::ade6*^+^ in the *ade6-DN/N* genetic background formed darker colonies. Note that the colony colors of canonical heterochromatin mutants (i.e. *clr4∆*) in the *ade6-m210* background were faint pink, and are not as white as those of strains with the native *ade6*^+^ allele in the endogenous *ade6* locus. **Figure S12.** Reintroduction of the R33C mutation phenocopied the original *A4* isolate. **(A)** Schematic diagram showing the introduction of the mutation (R33C) found in the original *A4* isolate. **(B)** Cells were streaked on normal YES plates (YES) and YES containing limited amount of adenine (Low adenine), and incubated at 30°C for four days. In HKM-1565 and HKM-1618, the G418-resistant *kanMX* cassette was located upstream of the *hsp90* promoter. Both strains were generated by introducing *kanMX*-containing DNA fragments with or without the R33C mutation into the genome of the wild-type strain HKM-1100. **Figure S13.** Silver staining of immunoprecipitated proteins that were analyzed by mass spectrometry. Immunoprecipitates from indicated cells were separated by SDS-PAGE and silver-stained (see Methods). Each lane in the gel was sliced as indicated by red lines. Proteins in each gel piece were analyzed by nano-liquid chromatography tandem mass spectrometry. Black arrows indicate visible protein bands that are absent or barely seen in the untagged control (no-tag).

**Additional file 2: Table S1.** Strains used in this study. **Table S2.** Primers used in this study. **Table S3.** Transcription related factors detected in massspectrometry analysis. **Table S4.** List of candidate genes for silencing factors suggested by massspectrometry.

## Abbreviations

ARC: Argonaute small interfering RNA chaperone
ER: endoplasmic reticulum
5-FOA: 5-fluoroorotic acid
Hsp: heat-shock molecular chaperone protein
Hsp40: heat-shock protein 40
Hsp70: heat-shock protein 70
Hsp90: heat-shock protein 90
H3K9me: methylation of histone H3 at Lys-9
RNAi: RNA interference
RITS: RNA-induced transcriptional silencing
siRNA: small interfering RNA
