# Supplementary material for: RNAi-dependent heterochromatin assembly in fission yeast Schizosaccharomyces pombe requires heat-shock molecular chaperones Hsp90 and Mas5
Source: Epigenetics Chromatin. 2018 Jun 4;11:26. doi: 10.1186/s13072-018-0199-8 (PMC5985592; doi:10.1186/s13072-018-0199-8)

Additional file 1: Figure S1

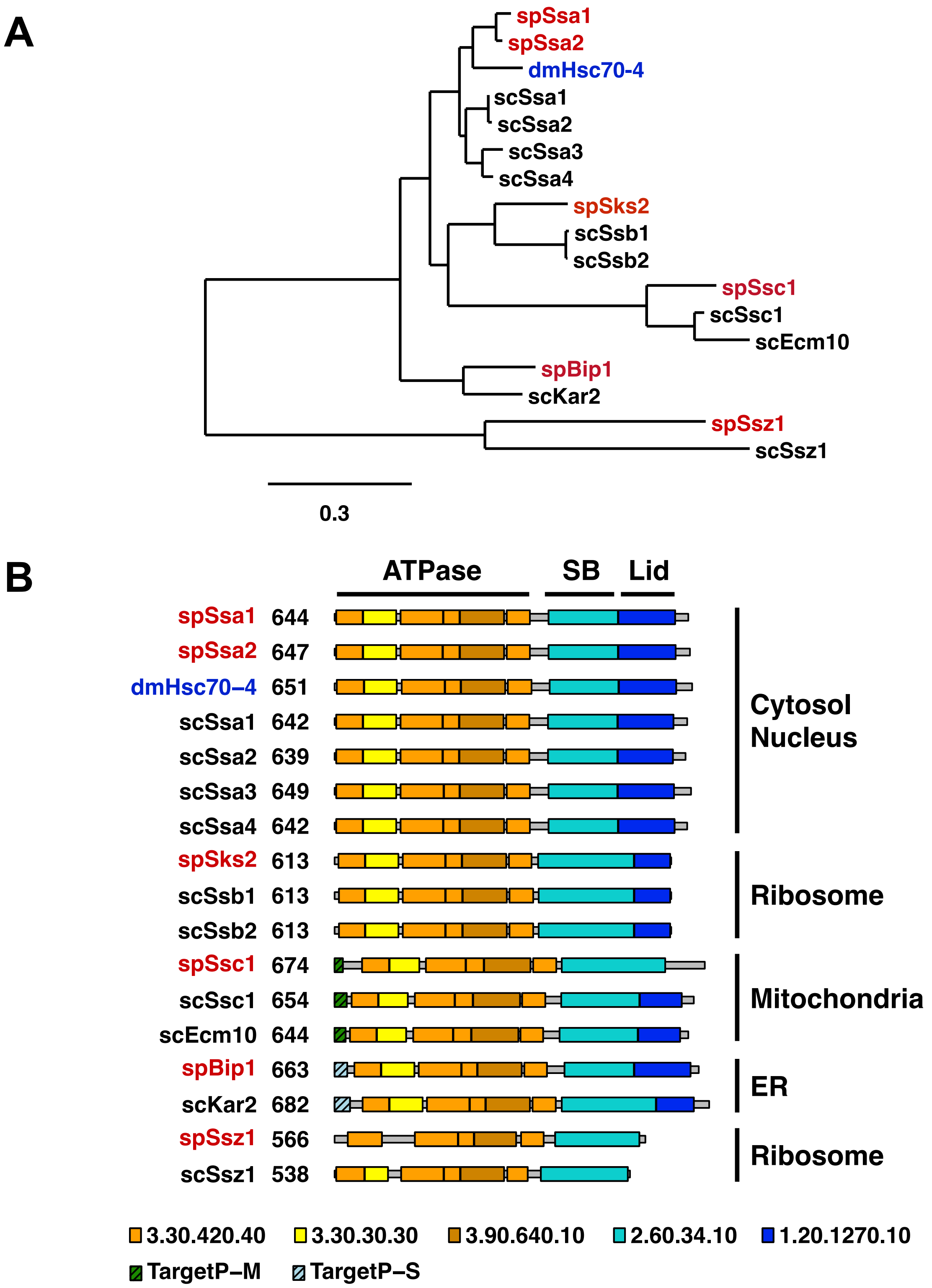

Additional file 1: Figure S2

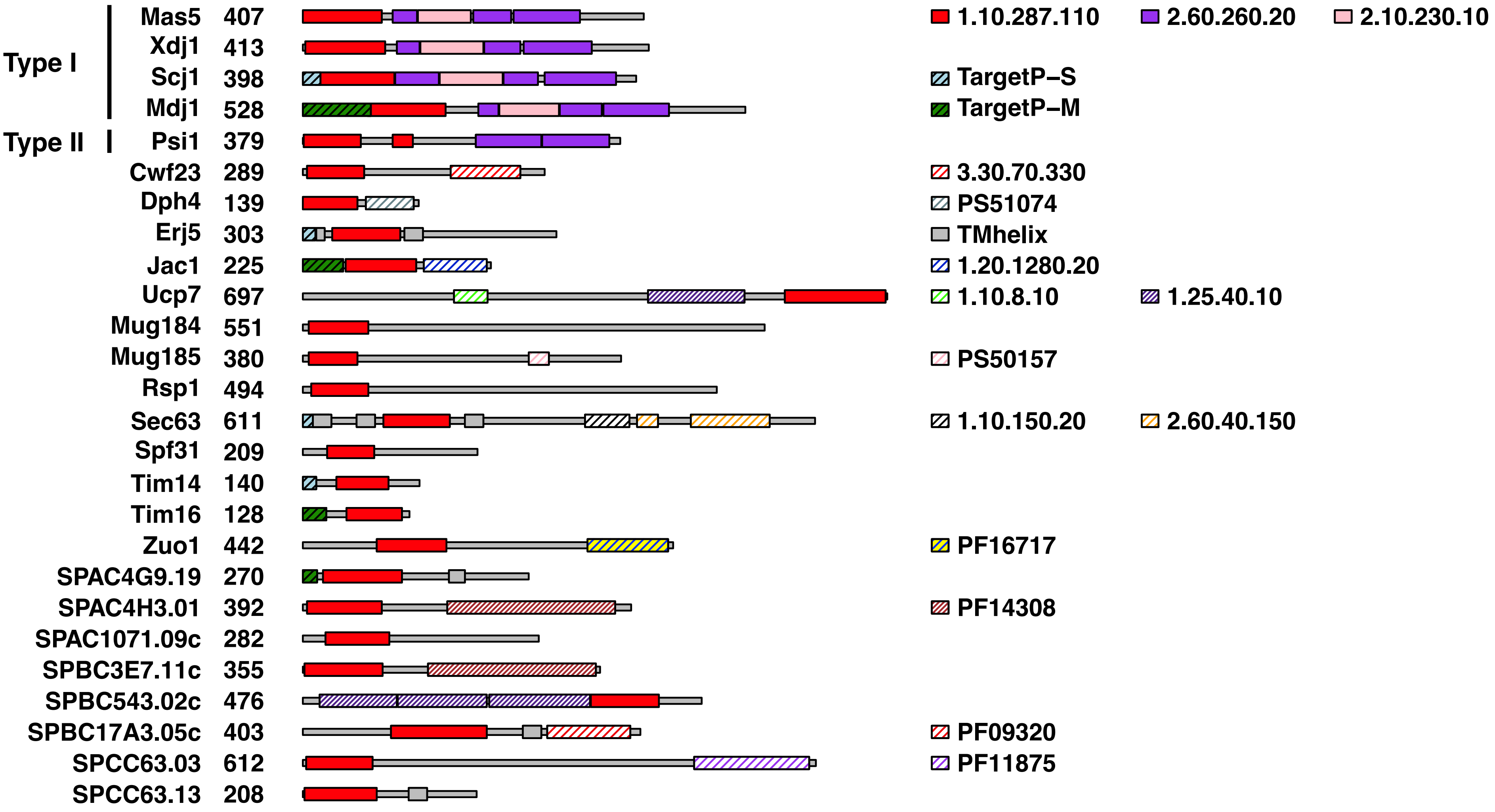

Additional file 1: Figure S3

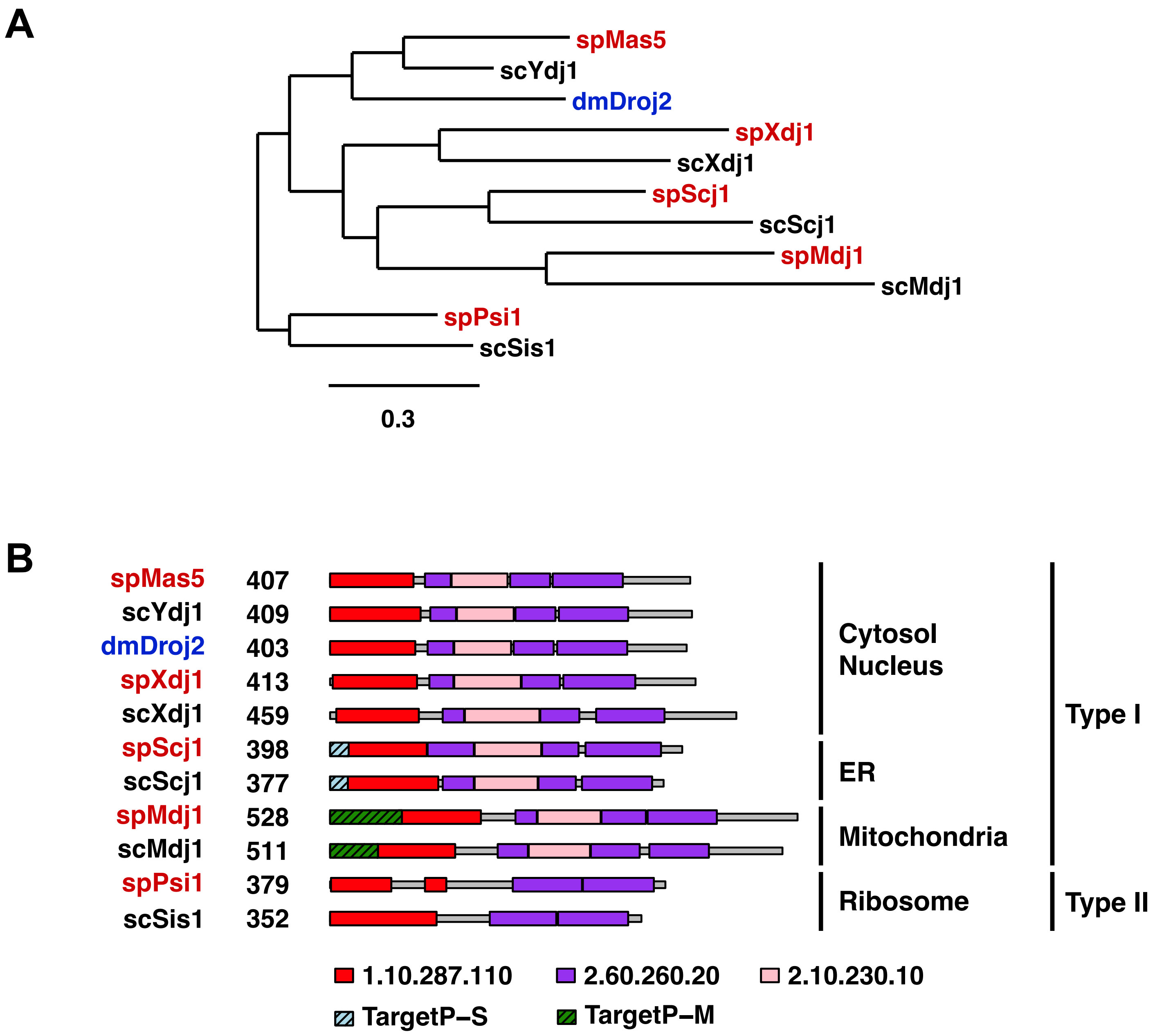

Additional file 1: Figure S4

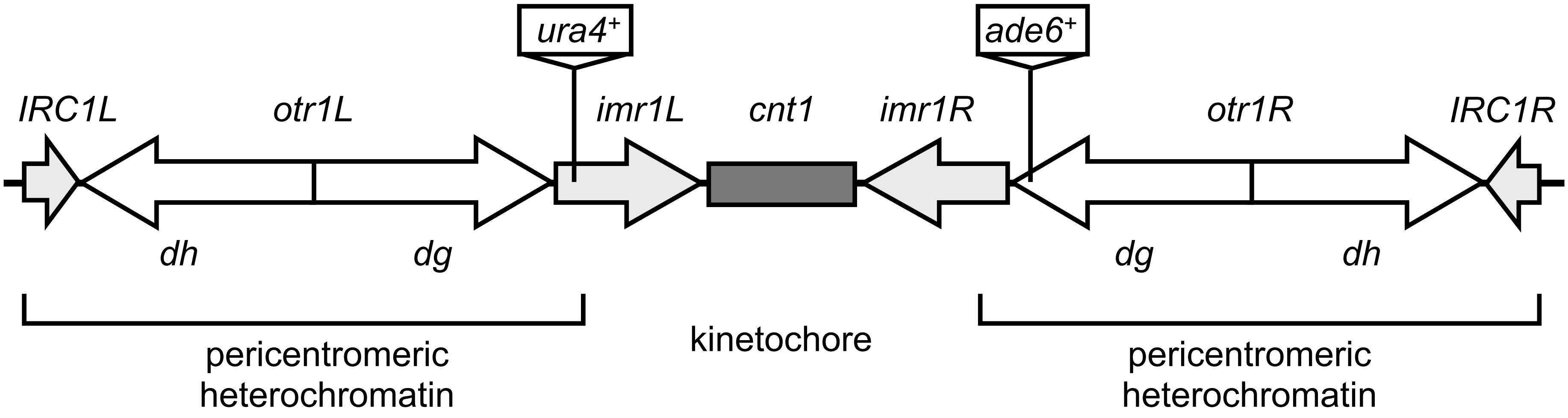

Additional file 1: Figure S5

Strain	*ade6* locus	
HKM-972	*ade6*$^{+}$	Wild-type
HKM-2299	*ade6-DN/N*	Wild-type
HKM-2306	*ade6-DN/N*	*∆clr4::hphMX*
HKM-2302	*ade6-DN/N*	*∆dcr1::hphMX*
HKM-2307	*ade6-DN/N*	*kanMX::hsp90-A4*
HKM-2304	*ade6-DN/N*	*∆mas5::kanMX*

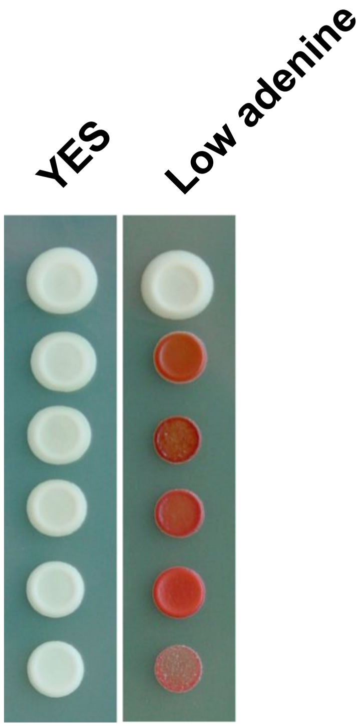

Additional file 1: Figure S6

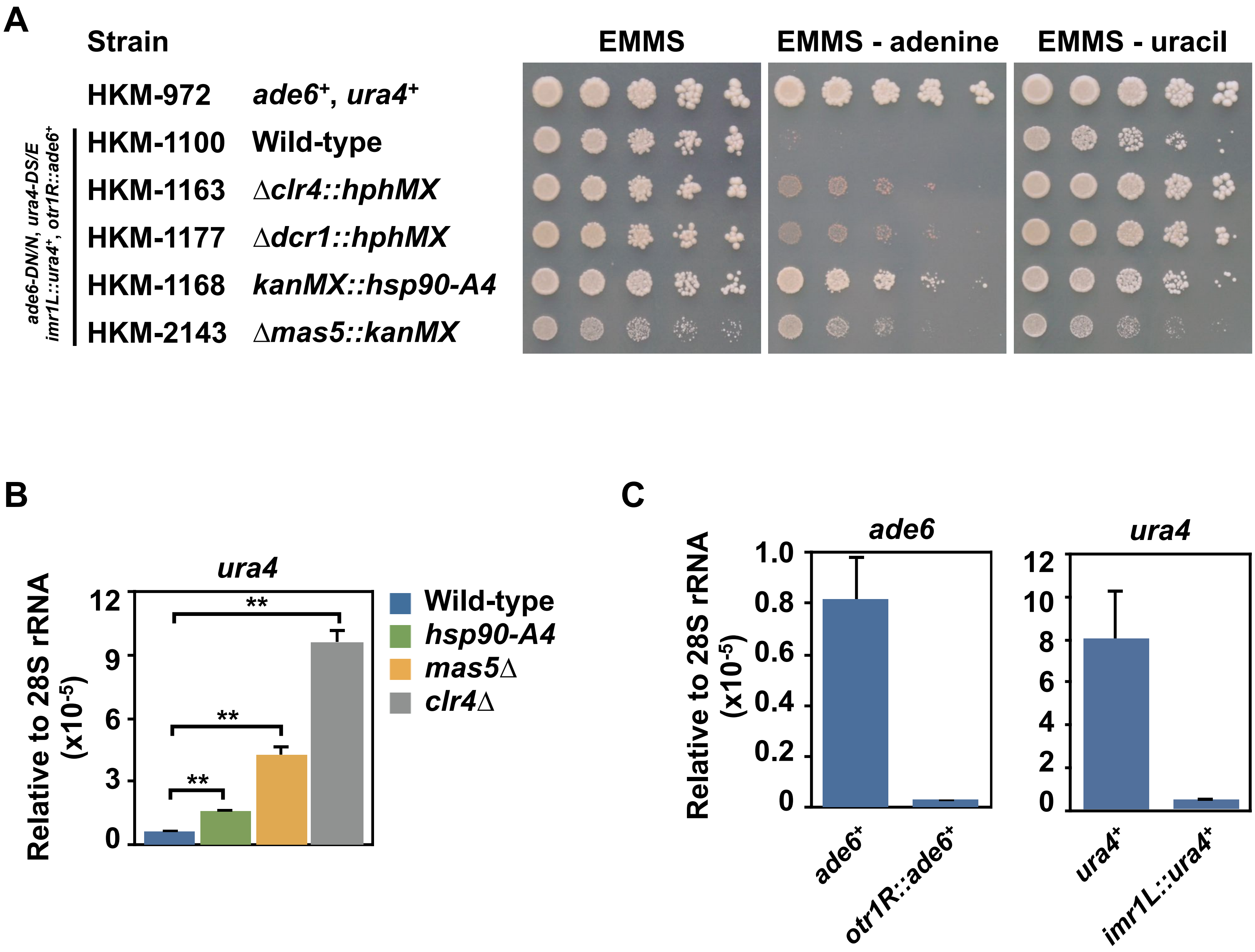

Additional file 1: Figure S7

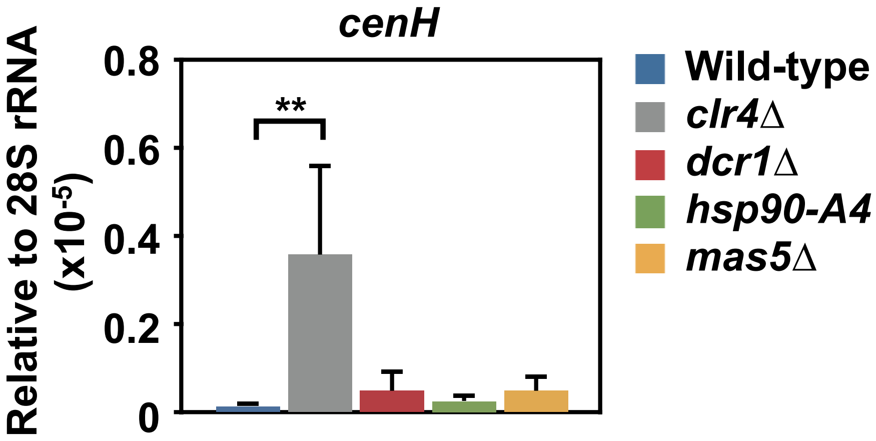

Additional file 1: Figure S8

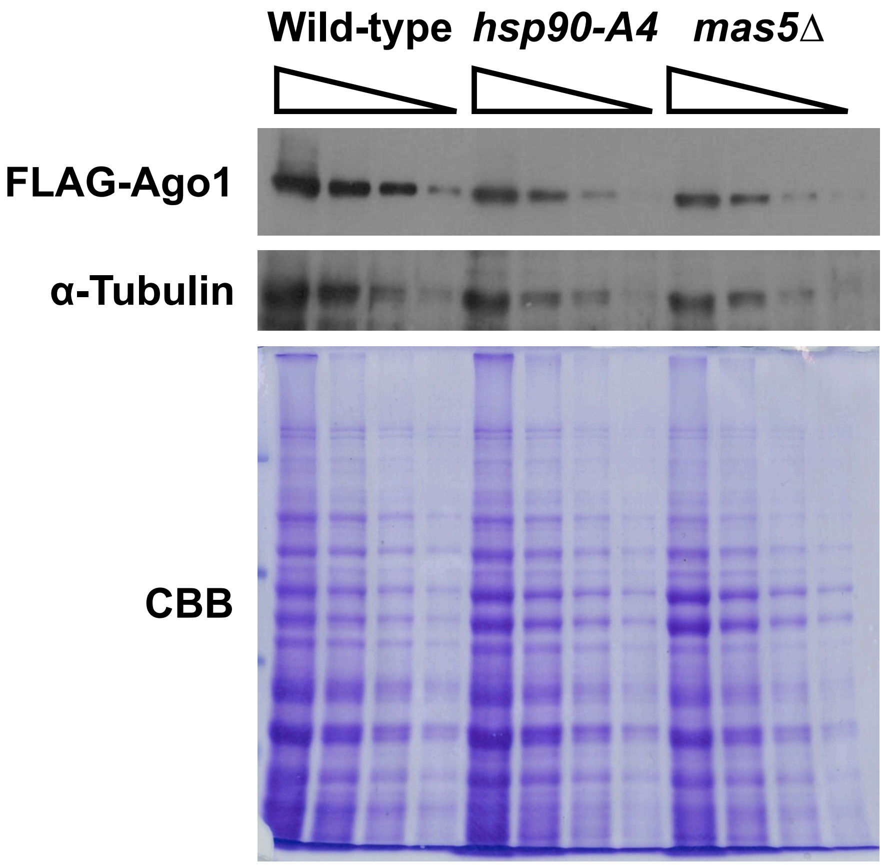

Additional file 1: Figure S9

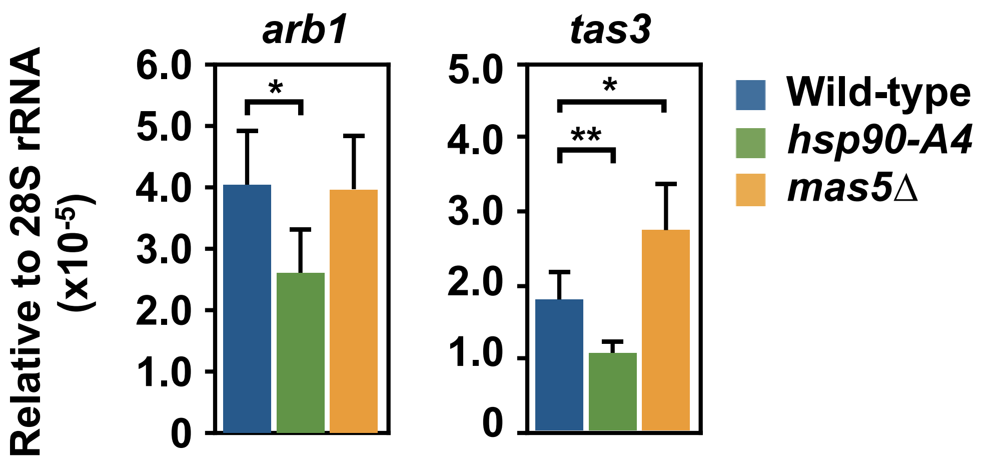

Additional file 1: Figure S10

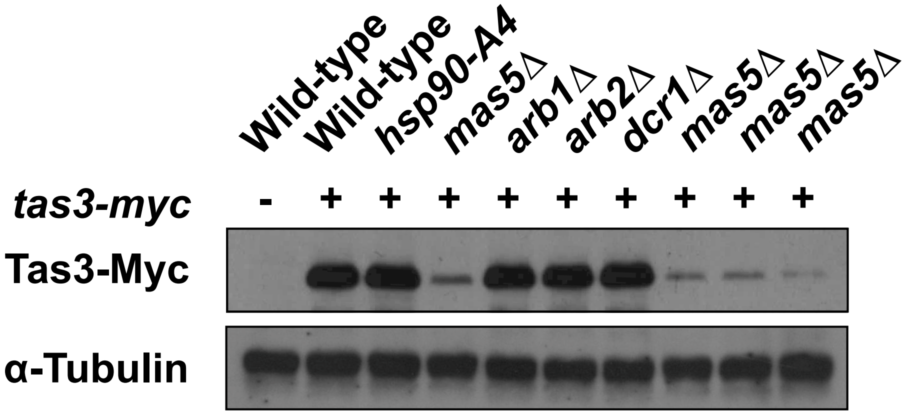

Additional file 1: Figure S11

Strain	*cen1* locus	*ade6* locus	*clr4* locus
HKM-972	*otr1R*$^{+}$	*ade6*$^{+}$	*clr4*$^{+}$
HKM-1200	*otr1R::ade6*$^{+}$	*ade6-m210*	*clr4*$^{+}$
HKM-1215	*otr1R::ade6*$^{+}$	*ade6-m210*	*clr4*Δ
HKM-1100	*otr1R::ade6*$^{+}$	*ade6-DN/N*	*clr4*$^{+}$
HKM-1163	*otr1R::ade6*$^{+}$	*ade6-DN/N*	*clr4*Δ

YES Low adenine

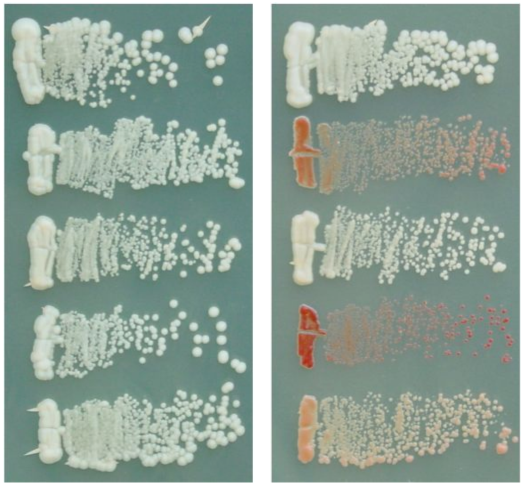

Additional file 1: Figure S12

A

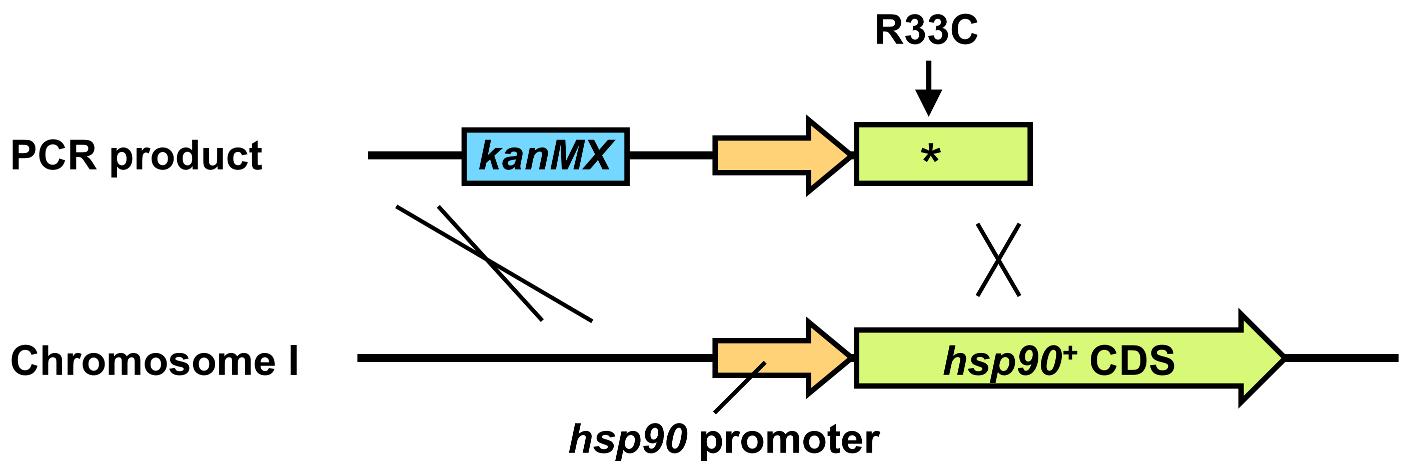

B

Strain	*clr4* locus	*hsp90* locus
HKM-1100	*clr4*$^{+}$	*hsp90*$^{+}$
HKM-1565	*clr4*$^{+}$	*kanMX::hsp90*$^{+}$
HKM-1258	*clr4*$^{+}$	*hsp90-A4*
HKM-1618	*clr4*$^{+}$	*kanMX::hsp90-A4*
HKM-1163	*clr4Δ::hphMX*	*hsp90*$^{+}$

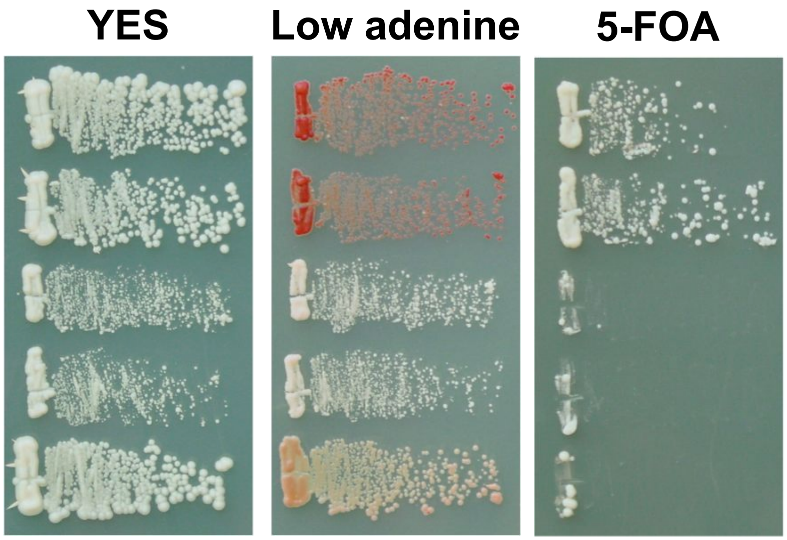

Additional file 1: Figure S13

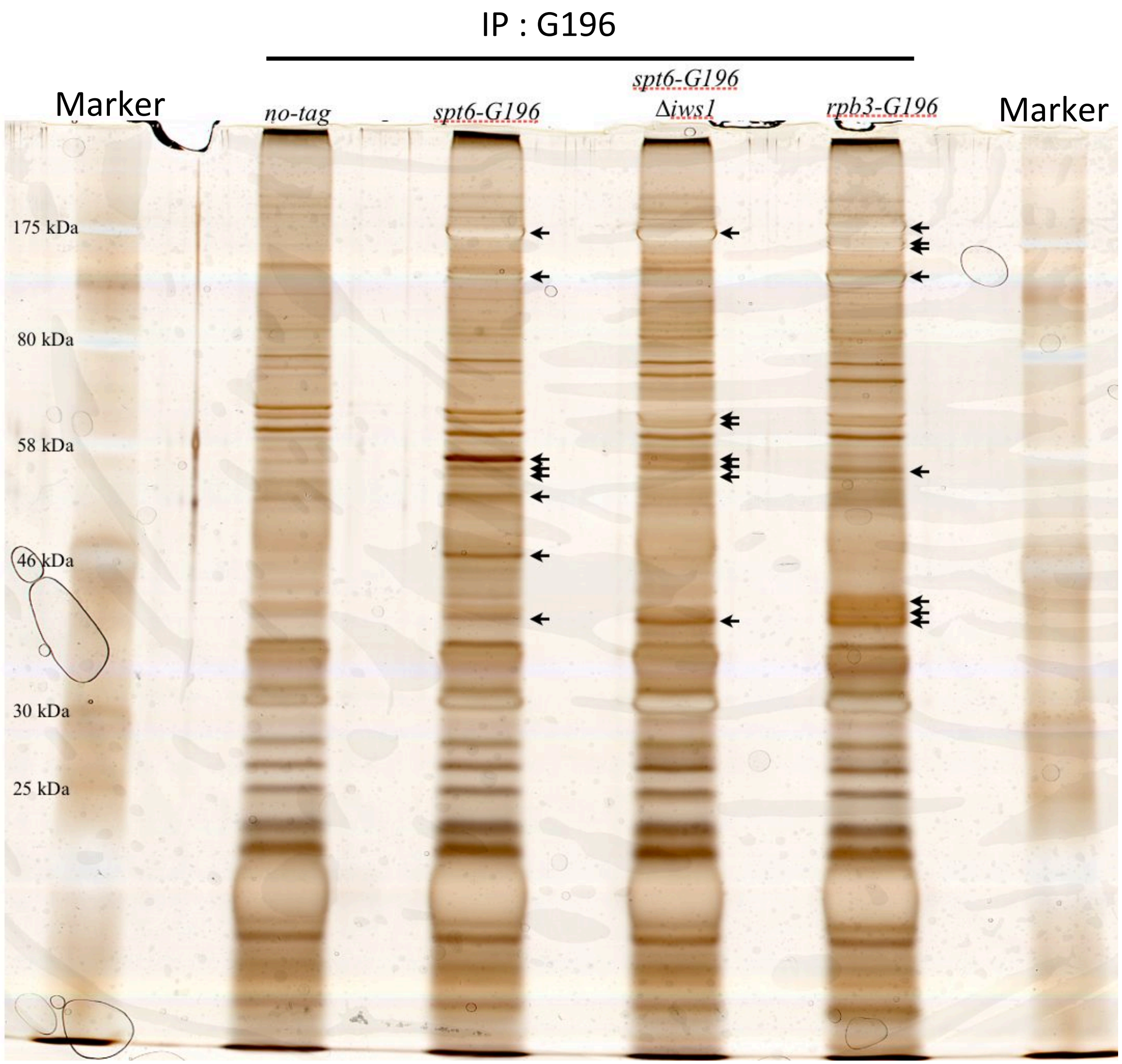

Supplement: Supplementary file 1 — Additional file 1: Figure S1. Fission yeast Hsp70 proteins and their homologs. (A) Phylogenic tree of Hsp70 proteins. Scale-bar unit indicates the number of amino acid substitutions per site. Names of proteins are associated with two-letter abbreviations and color-coded to indicate the species: “sp” for Schizosaccharomyces pombe (red), “sc” for Saccharomyces cerevisiae (black), and “dm” for Drosophila melanogaster (blue). (B) Domain structure of Hsp70 proteins. Protein names are depicted as in (A) and their amino acid lengths are shown. Protein domains are drawn as boxes according to the CATH-Gene3D classification. The N-terminal ATPase domains (ATPase) are composed of three internal domains that belong to the CATH superfamilies 3.30.420.40, 3.30.30.30, and 3.90.640.10. Substrate-binding domains (SB) belong to the 2.60.34.10 superfamily. The C-terminal lid domains (Lid) belong to the 1.20.1270.10 superfamily. Predicted localization signals for mitochondria (TargetP-M) and for the endoplasmic reticulum or beyond (TargetP-S) are shown as shaded boxes. Locations of the proteins in the cell are indicated. For clarity, D. melanogaster Hsp70 proteins other than Hsc70-4 are omitted. Figure S2. Hsp40 family proteins in fission yeast. Domain structure of all 26 fission yeast Hsp40 proteins are shown. Protein names and amino acid lengths are indicated. Protein domains are drawn as boxes according to the CATH-Gene3D, Pfam, and Prosite classifications. Predicted localization signals are shown as in Additional file 1: Figure S1. Predicted trans-membrane helix regions (TMhelix) are indicated as gray boxes. The DnaJ domains belong to the CATH superfamily 1.10.287.110. Type-I Hsp40 proteins are characterized by C-terminal (purple) and central (pink) domains that belong to the CATH superfamilies 2.60.260.20 and 2.10.230.10, respectively. The type-II Hsp40 protein Psi1 also contains the C-terminal domain but lacks the central domain. The other proteins are classified as the type-III [file 13072_2018_199_MOESM1_ESM.pdf]
